# The subgenus Monotarsobius in the Iberian Peninsula with a description of a new pseudo-cryptic species from Northern Spain revealed by an integrative revision of *Lithobius crassipes* L. Koch, 1862 (Chilopoda, Lithobiomorpha, Lithobiidae)

**DOI:** 10.3897/zookeys.681.12942

**Published:** 2017-06-21

**Authors:** Karin Voigtländer, Etienne Iorio, Peter Decker, Jörg Spelda

**Affiliations:** 1 Senckenberg Museum of Natural History Görlitz, Am Museum 1, 02826 Görlitz, Germany; 2 Groupe d’Étude des Invertébrés Armoricains, 5 rue du Général Leclerc, 44390 Nort-sur-Erdre, France; 3 Bavarian State Collection of Zoology, Münchhausenstraße 21, 81247 Munich, Germany

**Keywords:** COI, taxonomy, new species, pseudo-cryptic diversity, key, checklist

## Abstract

The widespread European centipede species Lithobius (Monotarsobius) crassipes L. Koch, 1862 was revised using an integrative approach incorporating sequence data and morphology. The partial mitochondrial cytochrome *c* oxidase subunit I (COI) barcoding gene was amplified and sequenced for 21 individuals from northern Spain, France and Germany as well as for individuals of three other species of the subgenus Monotarsobius Verhoeff, 1905. The dataset was used for molecular phylogenetic analysis and genetic distance determination. In addition, *Monotarsobius* specimens from more than 100 localities in northern Spain, France, and Germany were morphologically investigated. Both morphological and molecular data indicate that specimens from the Navarre and Gipuzkoa provinces, northern Spain, represent a distinct pseudo-cryptic species, only differing in some minor characters from *L. crassipes*. The new species L. (Monotarsobius) crassipesoides **sp. n.** is described and compared to *L. crassipes* in detail using morphology and morphometric statistics for body, head, and antennae length, number of ocelli and coxal pores, as well as the starting leg for legpair spines Vmt and DaP. The Iberian and European records of *L. crassipes* are discussed. The subspecies *L. crassipes morenoi* Garcia Ruiz, 2014 from Southern Spain is elevated to species as *L. morenoi* **stat. n.** A checklist, distribution map and key to all five species of *Monotarsobius* of the Iberian Peninsula are presented.

## Introduction

Currently 53 species and seven subspecies of the genus *Lithobius* Leach, 1814 are known from the Iberian Peninsula (Salinas 1990, Garcia Ruiz 2015, Iorio 2016, Iorio unpublished), of which 24 species and six subspecies are endemic to the region, which comprises continental Spain and Portugal. Of the 60 species-level taxa of Iberian Lithobius 48 species and six subspecies are in subgenus Lithobius, with four taxa in Monotarsobius Verhoeff, 1905 and two in subgenus Sigibius Chamberlin, 1913. *Monotarsobius* currently comprises the species *L. crassipes* L. Koch, 1862 and three other taxa endemic to Spain: *L. blascoi* Eason, 1991, *L. crassipes morenoi* Garcia Ruiz & Baena, 2014, and *L. osellai* Matic, 1968. This subgenus was reviewed by Serra (1982) for Iberia, together with a species from the Canary Islands and two species assigned to *Sigibius*. Beside the very few records of *L. blascoi*, *L. osellai* and *L. c. morenoi*, most *Monotarsobius* records are of *L. crassipes* (Meinert 1872, Attems 1952, Barace and Herrera 1980, Serra 1980, 1982).

Following a field trip to Navarre province in northern Spain in 2009 (see Material and methods), we noted that specimens of *L. crassipes* from the area differed slightly in morphology from other European *L. crassipes*. Fresh and museum material of *L. crassipes* from several localities in northern Spain, France and Germany was then studied in order to clearly delimit the new Spanish *L. crassipes*-like species, with integrated use of phylogenetic data, classical morphology and morphometric statistics. Here we also elevate *L. crassipes morenoi* to full species status and present an annotated list and updated key to species of *Monotarsobius* on the Iberian Peninsula. Finally, the implications on species distribution of *L. crassipes* on the Iberian Peninsula as well as in other parts of Europe are discussed.

## Material and methods

**Specimen collecting and preservation.** Material of *Lithobius crassipesoides* sp. n. was hand-collected during a collecting trip in Navarre province (Spain) in 2009. Participants and collectors (collectively referred to as FT2009) were Karin Voigtländer, Hans Reip, Norman Lindner, Desmond Kime, Helen Read, Henrik Enghoff, Paul Richards, Steve Gregory, and Per Djursvoll.

Additional material (in ethanol or mounted at slides) was borrowed from the Museum of Zoology of the University of Navarra (MZNA).

Material of *Lithobius crassipes* from various regions in Germany in the Senckenberg Museum of Natural History Görlitz (SMNG) was also investigated, both old material and from recent collections by N. Lindner. The studied French *L. crassipes* were collected from various regions in France and are partly kept in the private collection of E. Iorio, a minor part being also kept in the Groupe d’Étude des Invertébrés Armoricains (GRETIA) collection in Nort-sur-Erdre, France. The French specimens used for DNA extraction came from the forest of the Gȃvre (collected by E. Iorio), in the municipality of Le Gâvre in Loire-Atlantique department and are kept in the SMNG collection. All material is preserved in 70 % or 96 % ethanol (DNA vouchers) respectively. During the German Barcoding of Life project Myriapoda (Decker et al. 2017, Wesener et al. 2015, 2016) 22 specimens were collected for sequencing from various localities in Germany, but also from eastern France, Austria and Wales. The vouchers are deposited in the Zoologische Staatssammlung München (ZSM).

Sixty-four specimens of *L. crassipesoides* sp. n. from Spain were morphologically studied. For *L. crassipes* 55 specimens from 36 localities in Germany and 131 specimens from 60 localities in France were studied (see supplementary Table 1 for localities and collection numbers). Only adult specimens were used for morphological characters. Characters mentioned in the description of *L. crassipesoides* sp. n. are very similar or like in *L. crassipes*, unless stated otherwise. All specimens for molecular study were investigated for morphology prior to DNA extraction.

**Table 1.** Species, localities, GenBank accession numbers, and repository accession numbers for all specimens analyzed.

Species	Locality	GenBank Acc. No.	Voucher
**Ingroup**			
*L. crassipesoides* sp. n.	Spain, Navarra, Leitza, between area „Ustarleku“ and „Karobieta“	MF123704	SMNG VNR017128-8
*L. crassipesoides* sp. n.	Spain, Navarra, Leitza, between area „Ustarleku“ and „Karobieta“	MF123705	SMNG VNR017129-3
*L. crassipesoides* sp. n.	Spain, Navarra, Leitza, near road NA 4150	MF123706	SMNG VNR017131-3
*L. crassipesoides* sp. n.	Spain, Spain, Navarra, Leitza, near road NA 4150	MF123707	SMNG VNR017131-4
*L. crassipesoides* sp. n.	Spain, Navarra, Sierra de Aralar, S Errazkin	MF123708	SMNG VNR017135-4
*L. crassipesoides* sp. n.	Spain, Gipuzkoa, Onati, Aizkorri-Aratz	MF123709	SMNG VNR017139-5
*L. crassipes*	Germany, Baden-Württemberg, Bad Urach	KX458777	GBOL14972
*L. crassipes*	Germany, Bavaria, Höllental	MF123703	SMNG VNR17287-1
*L. crassipes*	Germany, Bavaria, Kelheim	JQ350449	BCZSMMYR00443
*L. crassipes*	Germany, Bavaria, Klausenhöhle	KX458796	GBOL11199
*L. crassipes*	Germany, Bavaria, Poppendorf	KX458752	GBOL12250
*L. crassipes*	Germany, Bavaria, Eichstätt	KX458625	GBOL14992
*L. crassipes*	Germany, Hesse, Göttingen	JQ801572.1	
*L. crassipes*	Germany, Lower Saxony, Soltau	KX458601	GBOL11898
*L. crassipes*	Germany, Saxony-Anhalt, Bollenkopf	MF123701	SMNG VNR17291-1
*L. crassipes*	Germany, Schleswig-Holstein, Bad Segeberg	KX458716	GBOL12308
*L. crassipes*	France, Haut-Rhin, Sewen	KX458631	GBOL11870
*L. crassipes*	France, Haut-Rhin, Thann	KX458674	GBOL11887
*L. crassipes*	France, Moselle, Ballon d`Alsace	KX458683	GBOL11895
*L. crassipes*	France, Loire-Atlantique, Le Gâvre	MF123710	SMNG VNR17281-1
*L. crassipes*	Great Britain, Wales, Newbridge	KX458753	GBOL11881
*L. curtipes*	Germany, Bavaria, Neuschönau	KX458647	GBOL12396
*L. curtipes*	Germany, Bavaria, Hengersdorf	KX458722	GBOL14991
*L. curtipes*	Germany, Saxony-Anhalt, Elbingerode	KX458653	GBOL11899
*L. curtipes*	Germany, Saxony-Anhalt, Elbingerode	KX458595	GBOL11875
*L. curtipes*	Great Britain, Wales, Groesfaen Woods	KX458652	GBOL11815
*L. austriacus*	Germany, Bavaria, Grafenau	KX458587	GBOL11194
*L. austriacus*	Germany, Bavaria, Grafenau	KX458687	GBOL14980
*L. austriacus*	Germany, Bavaria, Karlsfeld	KX458792	GBOL11829
*L. austriacus*	Germany, Bavaria, Spiegelau	KX458633	GBOL12311
*L. austriacus*	Germany, Saxony, Oybin	KX458666	GBOL12326
*L. austriacus*	Austria, Upper Austria, Sankt Wolfgang	JQ350447	BCZSMMYR00442
*L. forficatus*	Germany, Mecklenburg-Western Pomerania, Rügen	MF123702	SMNG VNR17150-2
**Outgroup**			
*E. cavernicolus*	Croatia	KF715050.1	CHP-416
*C. parisi*	Germany	KU497164.1	GBOL02712
*S. linearis*	Germany	KM491663.1	ZFMK-TIS-15771

**Illustrations.** For scanning electron micrographs (SEM), samples were dehydrated through an ethanol series, dried in a desiccator overnight, and mounted on aluminum stubs before being sputter coated with gold-palladium. SEM images were taken using a JEOL JSM-6510LV microscope, and samples were removed from stubs and returned to alcohol upon examination.

Preserved specimens were imaged with a Leica M165 C or Leica DM5500B stereo microscope and DFC295 camera. Focus-stacked images were assembled from 10–25 source images using the software package Leica V4.5. All images of the z-stacks are available online at VIRMISCO (www.virmisco.org, Christian et al. 2017).

All figures were later edited using Adobe Photoshop CS4. Maps were created with ArcGIS 10.

**DNA extraction and molecular analysis.** At the SMNG, DNA was extracted from 2–4 legs each of six specimens of *L. crassipesoides* sp. n., three specimens of *L. crassipes* and one specimen of *L. forficatus* (Linnaeus, 1758) (Table 1). Total genomic DNA was extracted using the Qiagen DNAeasy Blood & Tissue kit following the standard protocol except that tissue was incubated for 48 h. All specimens were later deposited in the collections of the SMNG.

Polymerase chain reaction (PCR) was used for amplifying the COI barcode fragment using the primer pair LCO1490 and HCO2198 (Folmer et al. 1994). The following thermocycling profile was used to amplify fragments of COI: predenaturation at 95° C for 1 min, 35 cycles of 40 s at 94° C, 40 s at 51° C, and 1 min at 72° C, final extension step for 5 min at 72° C. All PCR mixes had a total volume of 10 µL and contained 1 µL template, 0.1 mM of each primer, 4 × 0.15mM dNTPs [Peqlab], 1 × PCR Buffer containing 2 mM MgCl_2_ [Peqlab], and 0.2u Polymerase [Peqlab]. All fragments were sequenced in both directions by Biodiversity and Climate Laboratory Centre, Frankfurt, Germany.

As part of the GBOL project at ZSM, 11 specimens of *L. crassipes*, five specimens of *L. curtipes* C. L. Koch, 1847, and six *L. austriacus* (Verhoeff, 1937) were extracted and sequenced by the Canadian Centre for DNA Barcoding (CCDB, Guelph, Canada) using standardized, high-throughput DNA extraction, PCR amplification and bidirectional Sanger sequencing (http://www.ccdb.ca/resources.php). For PCR and sequencing, a primer cocktail (Hebert et al. 2004) was used. See also Wesener et al. (2015) and Wesener et al. (2016).

All 32 new sequences were deposited in GenBank (see Table 1 for accession numbers).

**Figure 1.**
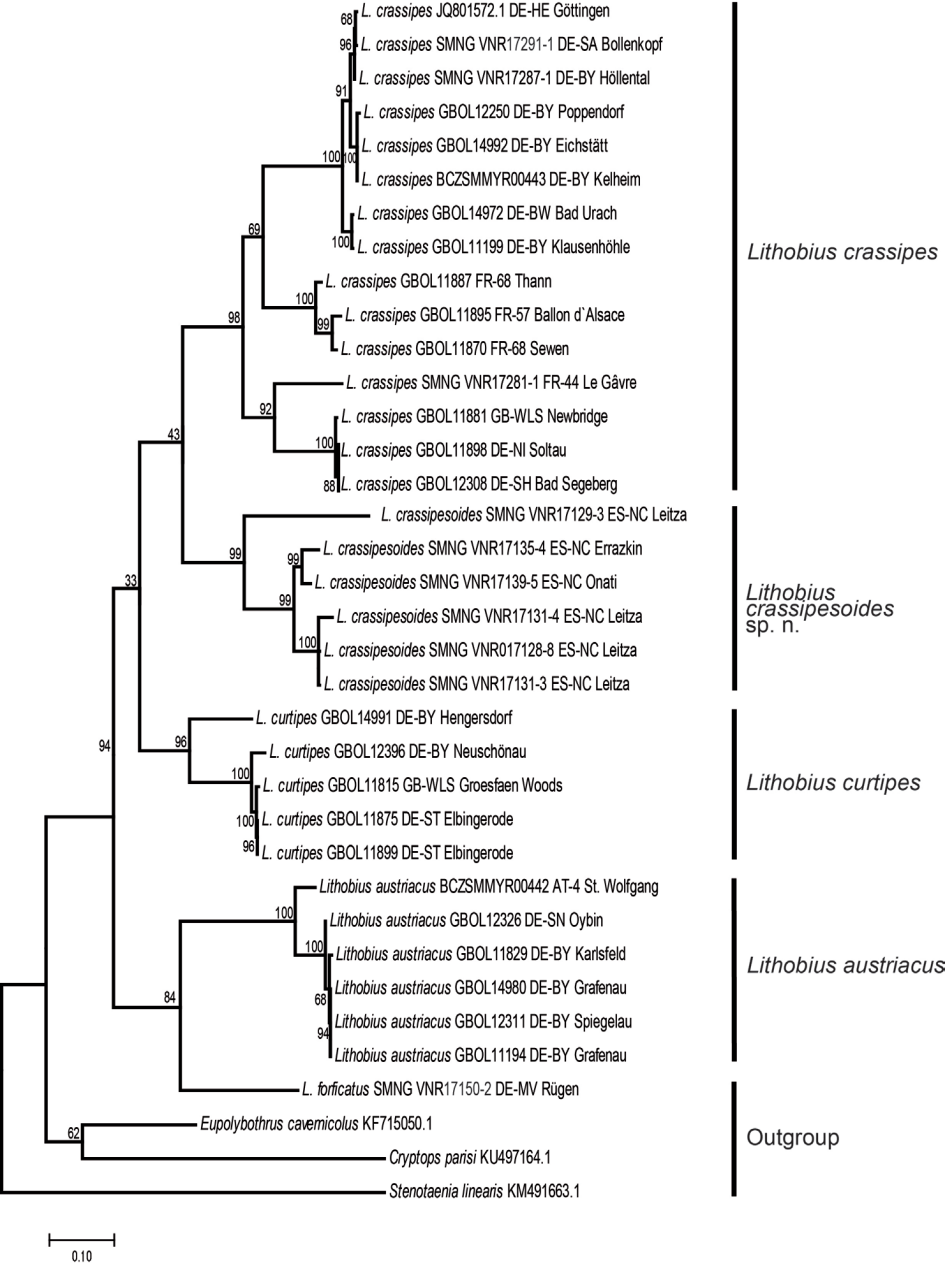
Results of the mitochondrial cytochrome c oxidase I (COI) analysis. Maximum likelihood tree, 1000 bootstrap replicates. Scale bar = substitutions per site.

**Alignment and phylogenetic analysis.** All obtained sequences were checked via Blast searches (Altschul et al. 1997); no contaminations were discovered. The sequences were aligned by hand in ClustalX ver. 1.83 (Chenna et al. 2003). One sequence for *L. crassipes* (Eitzinger et al. 2013) was downloaded from GenBank. As outgroups sequences *Eupolybothrus cavernicolus* Komerički & Stoev, 2013 (Stoev et al. 2013), *Stenotaenia linearis* (C.L. Koch, 1835) (Wesener et al. 2015) and *Cryptops parisi* Brölemann, 1920 (Wesener et al. 2016) were downloaded from GenBank (see Table 1 for accession numbers). The final dataset for the phylogenetic analysis included 26 sequences and the alignments consisted of 657 bp (COI mtDNA). To find the best substitution model, Modeltest as implemented in MEGA 7 (Kumar et al. 2016) was utilised. The lowest Bayesian Information Criterion score (BIC) was obtained for the Tamura–Nei model plus gamma distribution with indifferent sites (Tamura and Nei 1993) (BIC 11667.16). Maximum likelihood analyses were conducted in MEGA7 (Kumar et al. 2016). The tree with the highest log-likelihood (–5445.7387) is shown. Initial tree(s) for the heuristic search were obtained by applying the neighbour-joining method to a matrix of pairwise distances estimated using the maximum composite likelihood approach. A discrete gamma distribution was used to model evolutionary rate differences among sites (five categories (+*G*, parameter = 1.0671)). The tree (Fig. 1) is drawn to scale, with branch lengths measured in the number of substitutions per site. The bootstrap consensus tree inferred from 1000 replicates (Felsenstein 1985) is here used as the best estimate of the phylogeny of the analysed taxa (Fig. 1). The final trees were edited using Adobe Illustrator CS4. Mean uncorrected pairwise distances between terminals (transformed into percentages) were determined using MEGA7 (Kumar et al. 2016).

**Statistical analysis.** The software package PAST version 3.14 (Hammer et al. 2001) was used for analysis.

Box and jitter plots: For each sample, the 25–75 percent quartiles are drawn using a box. The median is shown with a horizontal line inside the box. The minimal and maximal values are shown with short horizontal lines (“whiskers”). Each value is plotted as a dot. To show overlapping points more clearly, they have been displaced using a random “jitter” value controlled by a slider.

Usually nonparametric statistics have been used, while parametric statistics have been restricted to interval data. Prior to the parametric t-test normality was tested using three statistical tests for normal distribution: The Shapiro-Wilk test (Shapiro and Wilk 1965), the Jarque-Bera-Test (Jarque and Bera 1987) and the Anderson-Darling-Test with significance estimated according to Stephens (1986). For the Jarque-Bera-Test and Anderson-Darling-Test also a significance test has been included based on Monte Carlo simulation, with 10,000 random values taken from a normal distribution has been included. If one of these normality tests rejected the null hypothesis of equal distribution only non-parametric statistics were used for the following tests.

In the xy graph of the canonical variate analysis (Figs 9–11) 95 % concentration ellipses have been plotted. Their calculation assumed normal distribution for the values of the discriminant factors. They estimate a region where 95 % of population points are expected to fall, i.e. they are not confidence regions for the mean.

The following characters have only been investigated in *L. crassipesoides* sp. n. from Spain and *L. crassipes* from Germany: the head length, the number of coxal pores on legpair 12–15 and the start of the ventral median spine on the trochanter.

**Terminology.** The terminology of external morphology follows Bonato et al. (2010).

The coloration as seen in alcohol material under a stereo microscope follows the colour terminology of Köhler (2012).

**The following abbreviations are used in the text and figures**:

**SMNG** Senckenberg Museum of Natural History

**ZSM** Zoologische Staatssammlung München

**MZNA** Museum of Zoology of the University of Navarra

**FT2009** participants in the Navarre field trip in 2009

**AL** antennae length

**BL** body length

**HL** head length

**LP** legpair

**C** coxa

**P** prefemur

**F** femur

**T** tibia

**t** trochanter

**Ts** tarsus

**a** anterior spine

**m** median spine

**p** posterior spine

**V** ventral

**D** dorsal

## Results

### Molecular analysis

The monophyly of both *Lithobius crassipesoides* sp. n. and *L. crassipes* is well supported with bootstrap values of 99 and 98 respectively (Fig. 1), as well as *L. curtipes* (96) and *L. austriacus* (99). A sister clade of *L. crassipesoides* sp. n. and *L. crassipes* is not supported (43), while all other groupings within the two species are well supported (Fig. 1). Intraspecific uncorrected p-distances range up to 16.8 % within *L. crassipesoides* sp. n., 16.3 % in *L. crassipes*, 12.6 % in *L. curtipes*, and 6.7 % in *L. austriacus*. Interspecific p-distances between *L. crassipesoides* sp. n. and *L. crassipes* range between 16.1 % and 21.2 %. Lowest interspecific distances between the other analysed *Monotarsobius* species are between *L. crassipes* and *L. curtipes* (16.6 %) and highest between *L. crassipes* and *L. crassipesoides* sp. n. (21.2 %). Uncorrected p-distances to the outgroup ranges from 18.3 % to 28.3 %.

**Figure 2.**
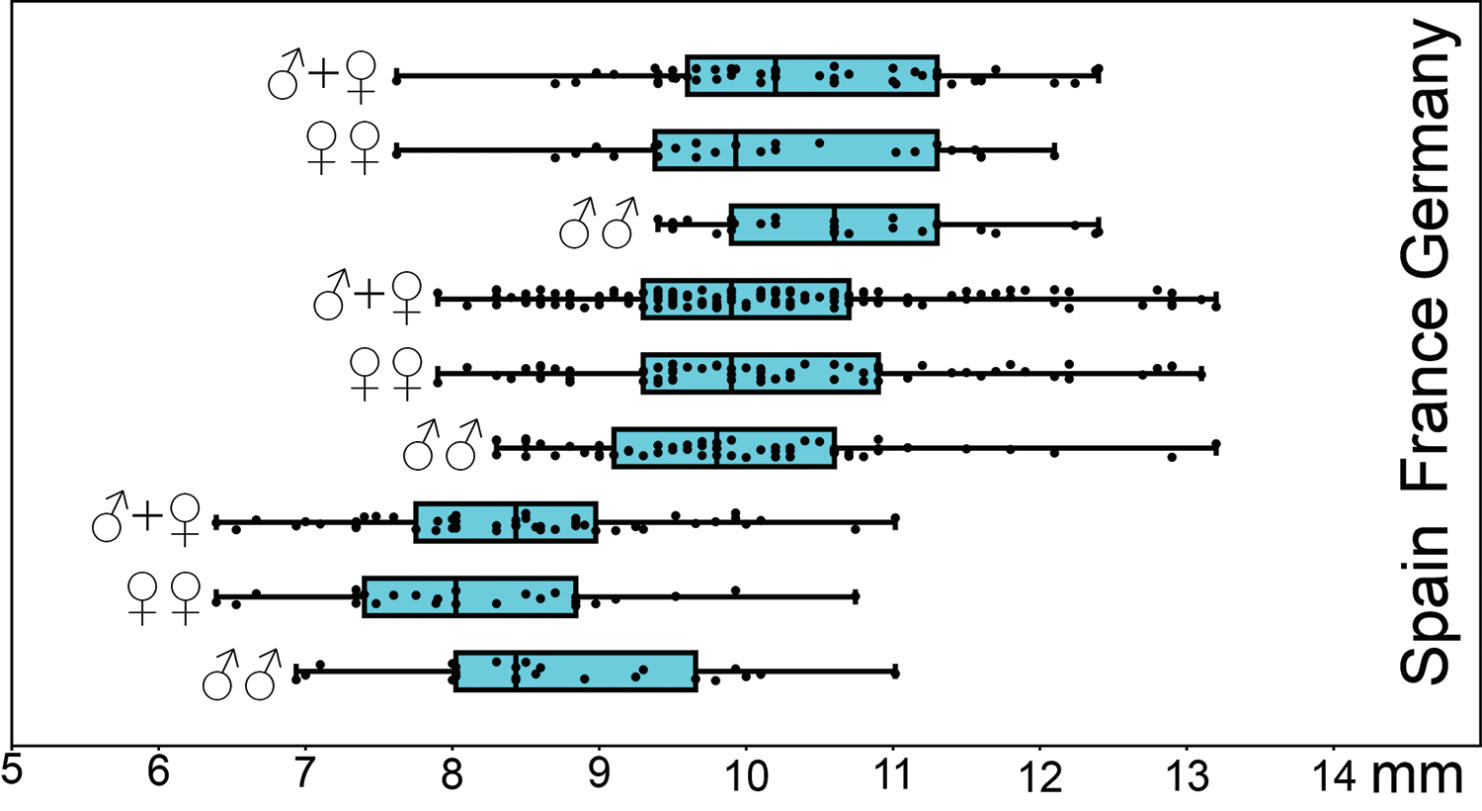
Body length (mm) of males and females of *Lithobius crassipesoides* sp. n. (Spain) and *L. crassipes* (Germany and France).

**Statistics of body length.** A comparison of jitter box plots shows that the specimens of *L. crassipesoides* sp. n. from Spain are usually smaller than those of *L. crassipes* (Fig. 2). The basic descriptive statistic parameters are given in Table 2.

**Table 2.** Body length (mm) of males and females of *Lithobius crassipesoides* sp. n. (Spain) and *L. crassipes* (Germany and France).

	*Lithobius crassipesoides*			*Lithobius crassipes*					
	Spain			France			Germany		
	♂♂	♀♀	♂+♀	♂♂	♀♀	♂+♀	♂♂	♀♀	♂+♀
N	24	26	50	62	69	131	27	25	52
Min	6.94	6.39	6.39	8.30	7.90	7.90	9.40	7.62	7.62
Max	11.02	10.74	11.02	13.20	13.10	13.20	12.40	12.10	12.40
Median	8.47	8.02	8.43	9.80	9.90	9.90	10.60	9.93	10.20
Mean	8.68	8.18	8.42	9.92	10.15	10.05	10.63	10.11	10.38
Stand. dev	1.04	1.04	1.06	1.06	1.31	1.20	0.90	1.12	1.04

The performed tests for normality (Table 3) showed significant differences from a normal distribution within the specimens from France, while there had been no significant deviation between the two sexes in the specimens from Spain and Germany. Based on the significance of the French samples it seemed to be wise to use non-parametric tests solely.

**Table 3.** Normality test for body length (mm) of males and females of *Lithobius crassipesoides* sp. n. (Spain) and *L. crassipes* (Germany and France). The p values below 0.05 are marked with bold letters.

	*Lithobius crassipesoides*			*Lithobius crassipes*					
	Spain			France			Germany		
	♂♂	♀♀	♂+♀	♂♂	♀♀	♂+♀	♂♂	♀♀	♂+♀
N	24	26	50	62	69	131	27	25	52
**Shapiro-Wilk W**									
p(normal)	0.387	0.729	0.690	**0.015**	**0.015**	**0.000**	0.089	0.467	0.416
**Anderson-Darling A**									
p(normal)	0.251	0.740	0.671	0.157	**0.021**	**0.002**	0.165	0.313	0.274
p(MCarlo)	0.261	0.761	0.687	0.165	**0.020**	**0.002**	0.167	0.319	0.289
**Jarque-Bera JB**									
p(normal)	0.794	0.718	0.637	**0.019**	0.146	**0.010**	0.405	0.796	0.897
p(MCarlo)	0.759	0.637	0.559	**0.023**	0.086	**0.018**	0.188	0.754	0.888

Using the Mann-Whitney-U-Test for mean, no significant differences in body length have been found between males and females of *L. crassipesoides* sp. n. from Spain (p=0.108) and between *L. crassipes* from France (0.433) and Germany (p=0.067). Therefore, it was justified to pool the samples. These pooled samples showed, that specimens of *L. crassipesoides* sp. n. are significantly shorter than specimens of *L. crassipes* from France (p<0.001) and Germany (p<0.001), while the two samples of *L. crassipes* do not significantly differ (p=0.081).

### Statistics of head length

**Figure 3.**
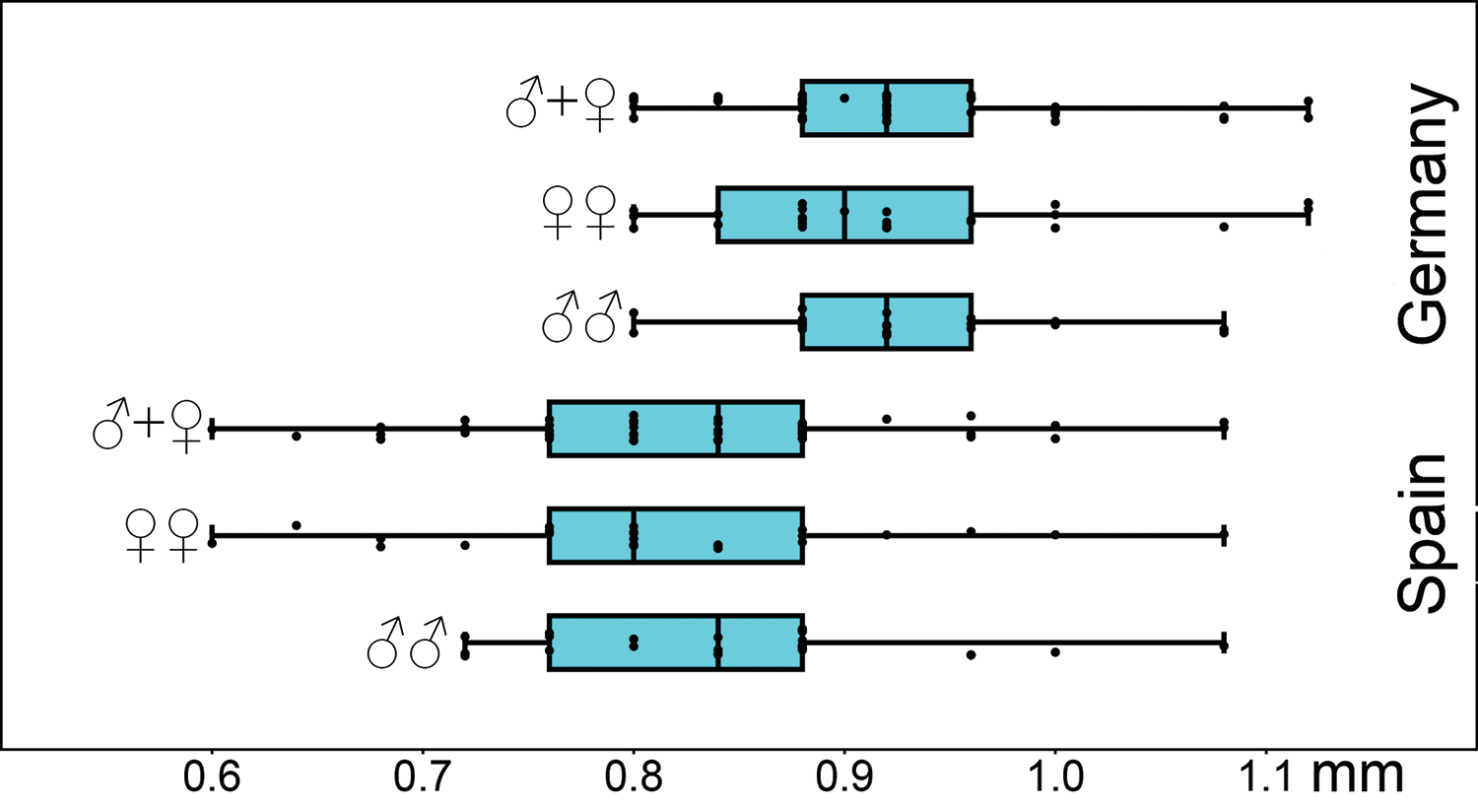
Head length (mm) of males and females of *Lithobius crassipesoides* sp. n. (Spain) and *L. crassipes* (Germany).

A comparison of jitter box plots shows, that the specimens of *L. crassipesoides* sp. n. from Spain usually have shorter heads than those of *L. crassipes* from Germany (Fig. 3). The basic descriptive statistic parameters are given in Table 4.

**Table 4.** Basic statistics of head length (in mm) in males and females of *Lithobius crassipesoides* sp. n. (Spain) and *L. crassipes* (Germany).

	*Lithobius crassipesoides*			*Lithobius crassipes*		
	Spain			Germany		
	♂♂	♀♀	♂+♀	♂♂	♀♀	♂+♀
N	24	26	50	25	27	52
Min	0.72	0.6	0.6	0.8	0.8	0.8
Max	1.08	1.08	1.08	1.08	1.12	1.12
Median	0.86	0.8	0.84	0.92	0.9	0.92
Mean	0.85	0.81	0.83	0.93	0.92	0.92
Stand. dev	0.09	0.11	0.10	0.07	0.09	0.08

The performed tests for normality (Table 5) showed significant differences from a normal distribution within the specimens from Germany in the Shapiro-Wilk W and Anderson-Darling A tests, while there had been no significant deviation between the two sexes in the specimens from Spain. Based on the significance of the German samples it seemed to be wise to use non-parametric tests solely.

**Table 5.** Normality test for head length (in mm) of males and females of *Lithobius crassipesoides* sp. n. (Spain) and *L. crassipes* (Germany). The p values below 0.05 are marked with bold letters.

	*Lithobius crassipesoides*			*Lithobius crassipes*		
	Spain			Germany		
	♂♂	♀♀	♂+♀	♂♂	♀♀	♂+♀
N	24	26	50	27	25	52
**Shapiro-Wilk W**						
p(normal)	0.105	0.572	0.241	**0.040**	**0.022**	**0.002**
**Anderson-Darling A**						
p(normal)	0.080	0.314	0.101	**0.016**	**0.037**	**0.001**
p(MCarlo)	0.076	0.316	0.100	**0.015**	**0.035**	**0.001**
**Jarque-Bera JB**						
p(normal)	0.585	0.705	0.675	0.563	0.236	0.130
p(MCarlo)	0.406	0.625	0.616	0.380	0.089	0.068

Using the Mann-Whitney-U-Test, no significant differences in head length have been found between males and females of *L. crassipesoides* sp. n. (p=0.105) and *L. crassipes* from Germany (0.655). The pooled samples show that head length was significantly shorter in specimens of *L. crassipesoides* sp. n. than in specimens of *L. crassipes* from Germany (p<0.001).

**Figure 4.**
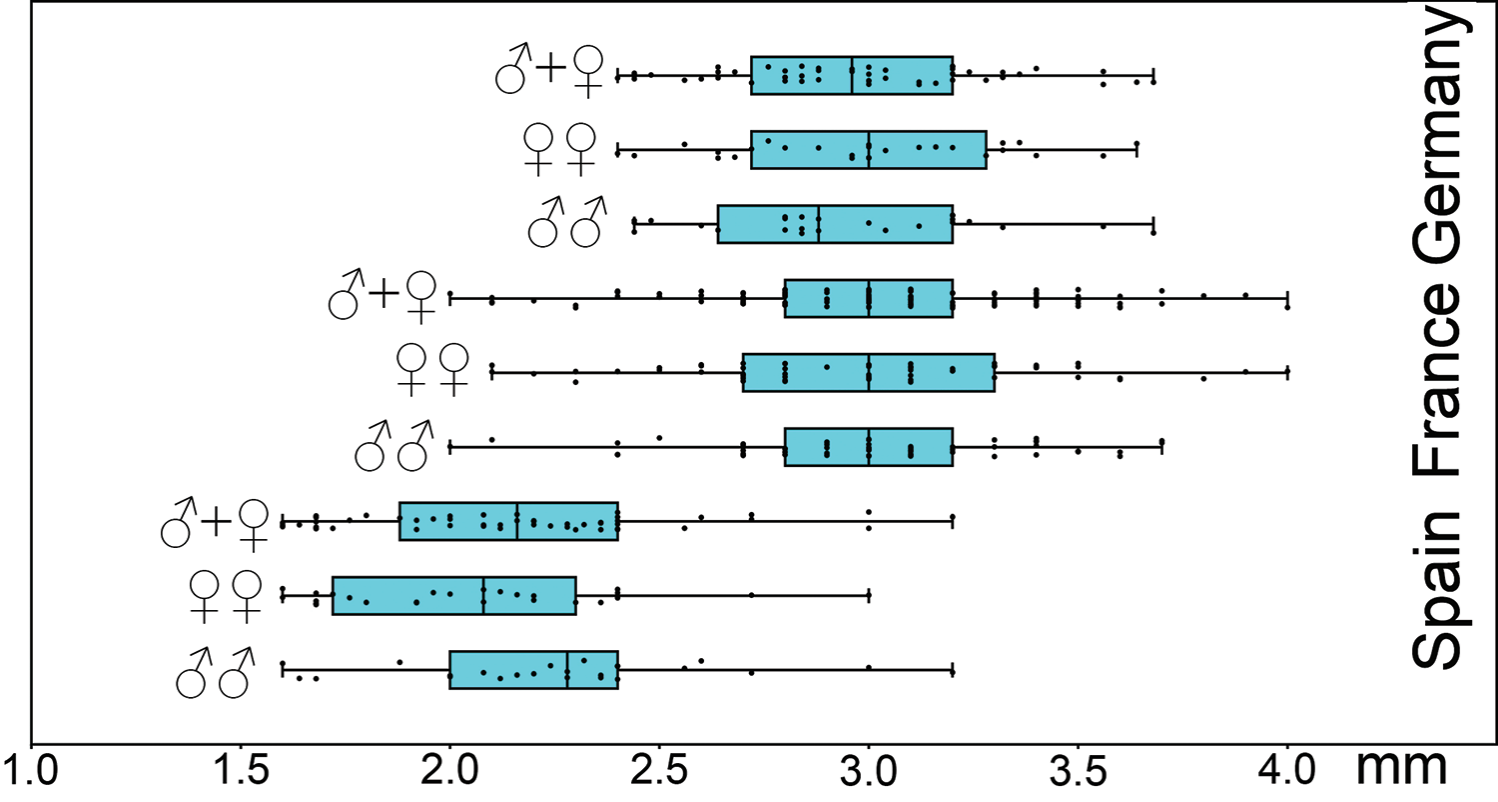
Antennae length (mm) of males and females of *Lithobius crassipesoides* sp. n. (Spain) and *L. crassipes* (Germany and France).

**Statistics of antennae length.** A comparison of jitter box plots shows, that the antennae of the specimens of *L. crassipesoides* sp. n. from Spain are usually smaller than those of *L. crassipes* (Fig. 4). The basic descriptive statistic parameters are given in Table 6.

**Table 6.** Antennae length (mm) of males and females of *Lithobius crassipesoides* sp. n. (Spain) and *L. crassipes* (Germany and France).

	***Lithobius crassipesoides***			***Lithobius crassipes***					
	**Spain**			**France**			**Germany**		
	♂♂	♀♀	♂+♀	♂♂	♀♀	♂+♀	♂♂	♀♀	♂+♀
N	24	26	50	59	59	118	25	27	52
Min	1.60	1.60	1.60	2.00	2.10	2.00	2.44	2.40	2.40
Max	3.20	3.00	3.20	3.70	4.00	4.00	3.68	3.64	3.68
Median	2.28	2.04	2.16	3.00	3.00	3.00	2.88	3.00	2.98
Mean	2.27	2.05	2.16	3.01	2.98	3.00	2.94	2.99	2.97
Stand. dev	0.39	0.36	0.38	0.36	0.42	0.39	0.34	0.33	0.33

The performed tests for normality (Table 7) showed low significant differences (p between 0.05 and 0.01) from a normal distribution only within pooled specimens from France and Spain, while there had been no significant deviation between the two sexes in the specimens from all countries and the pooled specimens from Germany. Based on the low significance of the French and Spanish samples it seemed to be wise to use non-parametric tests solely.

**Table 7.** Normality test for antennal length (mm) of males and females of *Lithobius crassipesoides* sp. n. (Spain) and *L. crassipes* (Germany and France). The p values below 0.05 are marked with bold letters.

	*Lithobius crassipesoides*			*Lithobius crassipes*					
	Spain			France			Germany		
	♂♂	♀♀	♂+♀	♂♂	♀♀	♂+♀	♂♂	♀♀	♂+♀
N	24	26	50	62	69	131	27	25	52
**Shapiro-Wilk W**									
p(normal)	0.486	0.079	**0.034**	0.083	0.700	0.195	0.307	0.869	0.327
**Anderson-Darling A**									
p(normal)	0.421	0.200	0.119	0.053	0.538	**0.043**	0.369	0.891	0.621
p(MCarlo)	0.424	0.203	0.116	0.054	0.554	**0.042**	0.378	0.896	0.645
**Jarque-Bera JB**									
p(normal)	0.726	0.299	0.291	0.253	0.932	0.804	0.739	0.737	0.569
p(MCarlo)	0.645	0.113	0.156	0.132	0.931	0.785	0.668	0.677	0.466

Using the Mann-Whitney-U-Test, only low significant differences in antennal length have been found between males and females of *L. crassipesoides* sp. n. from Spain (p=0.046) but not between *L. crassipes* from France (0.655) and Germany (p=0.555). The very low significance in specimens of *L. crassipesoides* sp. n. solely is interpreted as a stochastic effect and as there are no sex-specific differences in both of the *L. crassipes* samples they are pooled for comparison of the two species. These pooled samples showed that the antennae of specimens of *L. crassipesoides* sp. n. are significantly shorter than in specimens of *L. crassipes* from France (p<0.001) and Germany (p<0.001, while the two samples of *L. crassipes* do not significantly differ (p=0.632).

**Statistics of number of ocelli.** The number of ocelli was counted on each side of the head separately. For statistical evaluation, the average of the two sides has been used, as the separate measures are not independent samples (Lamprecht 1992). This approach also allows using ocelli numbers from a single side if the other was not countable.

**Figure 5.**
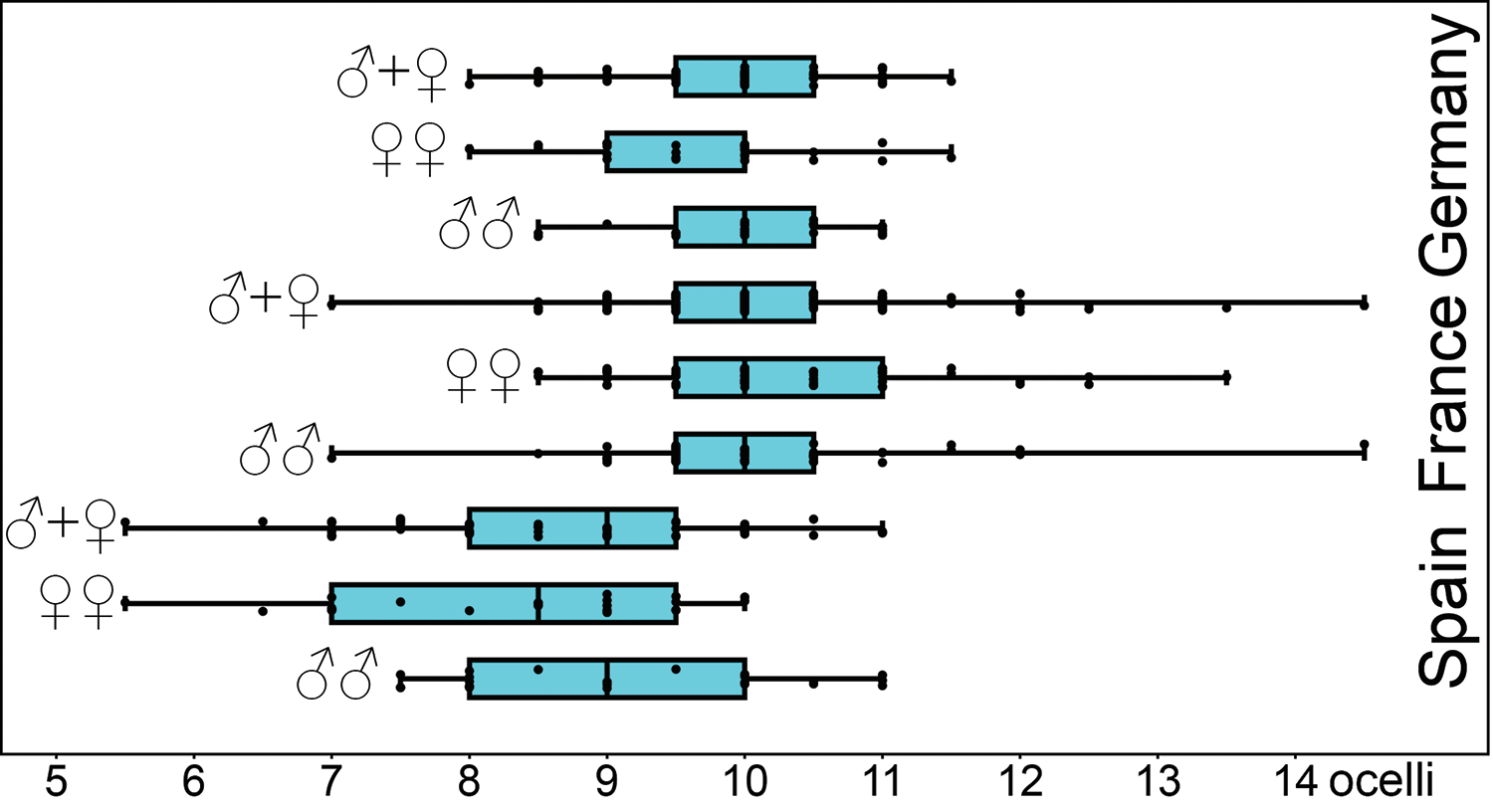
Number of ocelli in males and females of *Lithobius crassipesoides* sp. n. (Spain) and *L. crassipes* (Germany and France).

A comparison of jitter box plots shows that the specimens of *L. crassipesoides* sp. n. from Spain usually have a lower number of ocelli than specimens of *L. crassipes* from France and Germany (Fig. 5). The basic descriptive statistic parameters are given in Table 8.

**Table 8.** Number of ocelli of males and females of *Lithobius crassipesoides* sp. n. (Spain) and *L. crassipes* (Germany and France).

	*Lithobius crassipesoides*			*Lithobius crassipes*					
	Spain			France			Germany		
	♂♂	♀♀	♂+♀	♂♂	♀♀	♂+♀	♂♂	♀♀	♂+♀
N	23	24	47	59	66	125	25	27	52
Min	7.5	5.5	5.5	7	8.5	7	8.5	8	8
Max	11	10	11	14.5	13.5	14.5	11	11.5	11.5
Median	9	8.5	9	10	10	10	10	10	10
Mean	9.1	8.3	8.7	10.1	10.2	10.1	10.1	9.7	9.9
Stand. dev	1.2	1.2	1.3	1.0	1.0	1.0	0.7	0.8	0.8

Using the Mann-Whitney-U-Test, no significant differences in the number of ocelli have been found between males and females of *L. crassipesoides* sp. n. from Spain (p=0.063) and *L. crassipes* from France (0.438) and Germany (p=0.074). The pooled samples show that the number of ocelli in specimens of *L. crassipesoides* sp. n. is significantly lower than in specimens of *L. crassipes* from France (p<0.001) and Germany (p<0.001, while the two samples of *L. crassipes* do not significantly differ (p=0.293).

**Figure 6.**
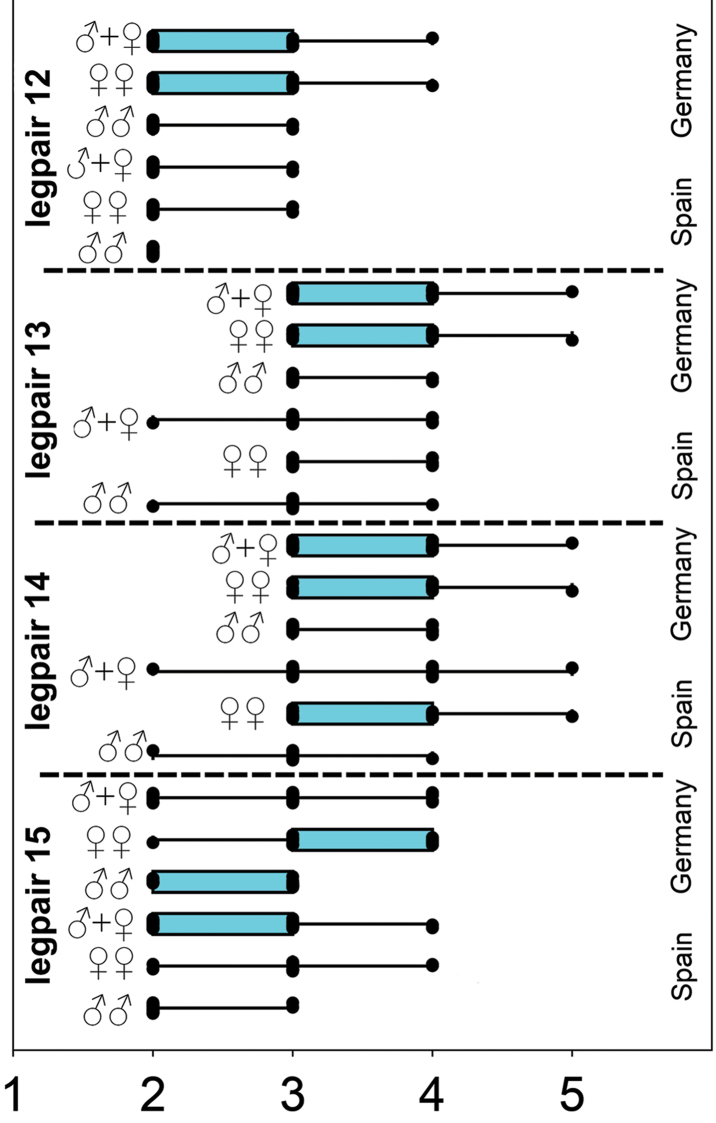
Number of coxal pores on legpair 12–15 in males and females of *Lithobius crassipesoides* sp. n. (Spain) and *L. crassipes* (Germany).

**Statistics of number of coxal pores.** A comparison of jitter box plots shows that specimens of *L. crassipesoides* sp. n. usually have a lower number of coxal pores on legpair 12 to 15 and that females usually have a higher number of coxal pores on them (Fig. 6). The basic descriptive statistic parameters are given in Table 9.

**Table 9.** Basic statistics of number of coxal pores on legpair 12–15 in males and females of *Lithobius crassipesoides* sp. n. (Spain) and *L. crassipes* (Germany).

	*Lithobius crassipesoides*			*Lithobius crassipes*		
	Spain			Germany		
	♂♂	♀♀	♂+♀	♂♂	♀♀	♂+♀
	legpair 12					
N	24	26	50	25	27	52
Min	2	2	2	2	2	2
Max	2	3	3	3	4	4
Median	2	2	2	2	2	2
Mean	2.00	2.08	2.04	2.12	2.41	2.27
Stand. dev	0.00	0.27	0.20	0.33	0.57	0.49
	**legpair 13**					
	♂♂	♀♀	♂+♀	♂♂	♀♀	♂+♀
N	24	26	50	25	27	52
Min	2	3	2	3	3	3
Max	4	4	4	4	5	5
Median	3	3	3	3	3	3
Mean	3.00	3.12	3.06	3.08	3.48	3.29
Stand. dev	0.29	0.33	0.31	0.28	0.58	0.50
	**legpair 14**					
	♂♂	♀♀	♂+♀	♂♂	♀♀	♂+♀
N	24	26	50	25	27	52
Min	2	3	2	3	3	3
Max	4	5	5	4	5	5
Median	3	3	3	3	4	3
Mean	3.00	3.42	3.22	3.20	3.74	3.48
Stand. dev	0.29	0.58	0.51	0.41	0.53	0.54
	**legpair 15**					
	♂♂	♀♀	♂+♀	♂♂	♀♀	♂+♀
N	24	26	50	25	27	52
Min	2	2	2	2	2	2
Max	3	4	4	3	4	4
Median	2	3	3	3	3	3
Mean	2.08	3.00	2.56	2.60	3.22	2.92
Stand. dev	0.28	0.40	0.58	0.50	0.51	0.59

In Table 10 the differences in coxal pore numbers between males and females as well as between the different species have been tested using the Mann-Whitney-U-Test. This test showed that females usually have a higher number of coxal pores than males. This had been more obvious in specimens of *L. crassipes* from Germany (significant p values in all legs) and also more obvious on the coxopleura of legpair 14 and 15 (significant p values in both species). Therefore, comparisons have to be made sex specific. These sex specific tests showed significant differences in the number of coxal pores on legpair 15 solely in males, while in females it was exactly legpair 15 that showed no significant p value. Pooled samples showed significant p values for coxal pore numbers in all legs, but this comparison is of doubtful value as it represents an average of the sex specific differences.

**Table 10.** Significance levels (p values) of differences in the numbers of coxal pores between males and females and pooled samples of *Lithobius crassipesoides* sp. n. (Spain) and *L. crassipes* (Germany). The p values below 0.05 are marked with bold letters.

	legpair 12	legpair 13	legpair 14	legpair 15
♂♂ (Spain) vs. ♀♀ (Spain)	0.179	0.197	**0.002**	**< 0.001**
♂♂ (Germany) vs. ♀♀ (Germany)	**0.037**	**0.003**	**< 0.001**	**< 0.001**
♂♂ (Spain) vs. ♂♂ (Germany)	0.087	0.343	0.06	**< 0.001**
♀♀ (Spain) vs. ♀♀ (Germany)	**0.01**	**0.008**	**0.041**	0.083
♂+♀ (Spain) vs. ♂+♀ (Germany)	**0.002**	**0.007**	**0.014**	**0.002**

**Statistics of legpair DaP spine.** The start of the anterior dorsal spine (DaP) at the prefemur has sometimes been checked on both sides, sometimes on one side solely, depending on the availability of intact specimens with at least one leg being complete on one side at a given legpair position. Again, the average value has been used for the statistical calculation if both values had been available. Due to the high number of specimens with missing legs the number of samples was much lower than in the previously processed characters, see Table 11 for basic descriptive statistic parameters.

**Table 11.** Legpair where anterior dorsal spine of prefemur (DaP) starts in males and females of *Lithobius crassipesoides* sp. n. (Spain) and *L. crassipes* (Germany and France). Abbreviation: n. p. = not present.

	*Lithobius crassipesoides*			*Lithobius crassipes*					
	Spain			France			Germany		
	♂♂	♀♀	♂+♀	♂♂	♀♀	♂+♀	♂♂	♀♀	♂+♀
N	24	26	50	36	34	70	25	26	51
Min	14	12	12	7,5	8	7.5	7	8	7
Max	15	n. p.	n. p.	11.5	11.5	11.5	11	13	13
Median	15	15	15	10	9.75	10	9	9	9
Mean	14.7	14.4	14.5	10.0	9.8	9.9	9.0	9.8	9.4
Stand. dev	0.5	1.0	0.8	0.9	0.7	0.8	1.2	1.4	1.4

**Figure 7.**
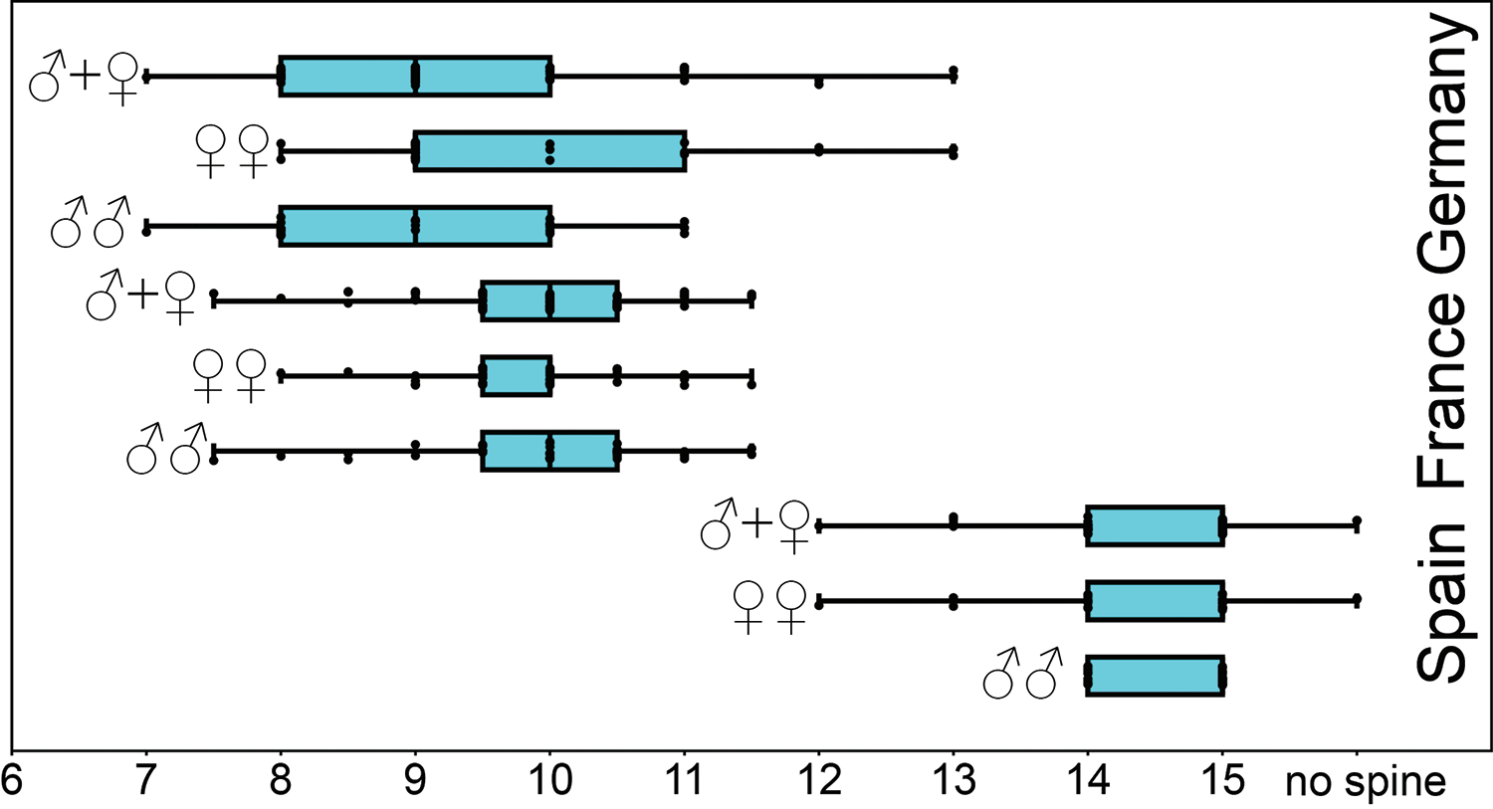
Start of the anterior dorsal spine of prefemur in males and females of *Lithobius crassipesoides* sp. n. (Spain) and *L. crassipes* (Germany and France). The DaP spine has been recorded on each leg of each specimen in French material: this explains the 0.5 graduation in this case (DaP being sometimes present on one side only).

A comparison of jitter box plots shows that the spine DaP usually starts later in specimens of *L. crassipesoides* sp. n. from Spain than in specimens of *L. crassipes* (Fig. 7), with an overlap at legpair 12 and 13 in females solely.

Using the Mann-Whitney-U-Test, no significant differences in the starting position of spine DaP have been found between males and females of *L. crassipesoides* sp. n. from Spain (p=0.209) and *L. crassipes* from France (p=0.340), while there was a low significance between males and females from Germany (p=0.024). The pooled samples show that spine DaP starts significantly later in specimens of *L. crassipesoides* sp. n. than in specimens of *L. crassipes* from France (p<0.001) and Germany (p<0.001). However, the two samples of *L. crassipes* differ significantly (p=0.001).

### Statistics of median ventral spine at the trochanter

The start of the median ventral spine at the trochanter (Vmt) has sometimes been checked on one side solely. The basic descriptive statistic parameters are given in Table 12.

**Table 12.** Basic statistics of legpair where median ventral trochanter spine (Vmt) starts in males and females of *Lithobius crassipesoides* sp. n. (Spain) and *L. crassipes* (Germany).

	*Lithobius crassipesoides*			*Lithobius crassipes*		
	Spain			Germany		
	♂♂	♀♀	♂+♀	♂♂	♀♀	♂+♀
N	24	26	50	25	27	52
Min	12	12	12	13	13	13
Max	14	14	14	14	14	14
Median	13	13	13	14	14	14
Mean	13.0	13.0	13.0	13.9	13.9	13.9
Stand. dev	0.6	0.3	0.5	0.3	0.4	0.3

**Figure 8.**
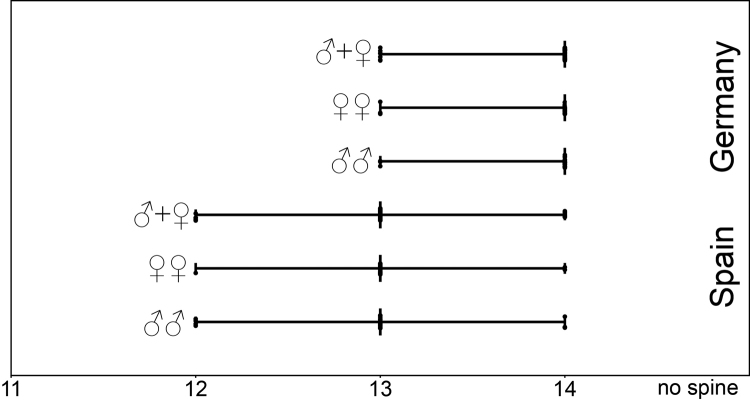
Legpair where the median ventral trochanter spine starts in males and females of *Lithobius crassipesoides* sp. n. (Spain) and *L. crassipes* (Germany).

A comparison of jitter box plots shows that the spine Vmt usually starts earlier in specimens of *L. crassipesoides* sp. n. from Spain than in specimens of *L. crassipes* (Fig. 8), with an overlap at legpair 13.

Using the Mann-Whitney-U-Test, no significant differences in the starting position of spine Vmt have been found between males and females of *L. crassipesoides* sp. n. (p=0.777) and *L. crassipes* from Germany (p=0.771). The pooled samples show that spine Vmt appeared significantly earlier in specimens of *L. crassipesoides* sp. n. than in specimens of *L. crassipes* from Germany (p<0.001).

### Canonical variate analysis

A canonical variate analysis (CVA, linear discriminant analysis with several groups) has been performed to improve the separation of the two species. As complete datasets are necessary for such a comparison only samples from Germany could be used to represent *L. crassipes*. All morphometric characters evaluated above have been used and sex specific groups have been defined. Additionally, the ratios of body length and antenna length to head length have been included.

**Figure 9.**
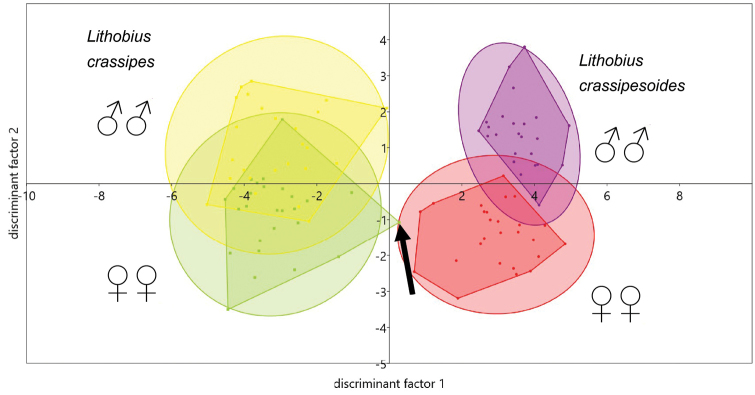
Canonical variate analysis of morphometric data showing factors one and two, using all morphometric characters of *Lithobius crassipesoides* sp. n. from Spain and *L. crassipes* from Germany. The specimen SMNG VNR15170-2 b with an extraordinary position within the CVA is marked with an arrow.

The CVA (Fig. 9) allowed a complete separation of all specimens, as shown by the convex hulls. Also, the 95 % concentration ellipses show a complete separation, although there was one female of *L. crassipes* (SMNG VNR 15170-2 b) that fell outside the concentration ellipse.

Although differences between the sexes are clearly visible, even CVA does not allow separating them completely. Three discriminant factors have been extracted, explaining 85.8 %, 12.1 % and 2.1 % of the observed variance.

The factor loadings of discriminant function one show that the start of spine DaP and the body length have the highest separating power for the two species (Table 13). Ocelli number, start of spine Vmt and antennal length are also of value. Discriminant factor one could be interpreted as a “size factor”. It is remarkable that head length and both ratios are of negligible influence. They are just redundant expressions of the size factor, which is best represented by body length.

**Table 13.** Eigenvalues and factor loading of the three extracted discriminant factors for the complete model. Highest factor loadings marked in bold letters and top values in larger fonts.

	Axis 1	Axis 2	Axis 3
Eigenvalue	10.6	1.49	0.26
Explanation of variance	85.8 %	12.1 %	2.1 %
Body length	-**0.303**	0.080	**0.579**
Antennae length	-**0.125**	0.063	0.147
Head length	-0.014	0.015	0.015
AL/HL	-0.097	0.026	0.132
BL/AL	0.067	-0.071	-0.004
Coxal pores LP 15	-0.064	-**0.316**	0.012
Coxal pores LP 14	-0.044	-0.187	0.157
Coxal pores LP 13	-0.037	-0.090	0.197
Coxal pores LP 12	-0.037	-0.062	0.145
Ocelli	-**0.170**	**0.262**	0.120
DaP	**0.804**	-**0.202**	**0.400**
Vmt	-**0.132**	0.017	-0.001

Discriminant factor two could be interpreted as a “sex factor”, with the highest factor loading on the number of coxal pores on legpair 15. Ocelli number and start of DaP are also of importance, while the numbers of coxal pores on the other legs are redundant characters.

**Figure 10.**
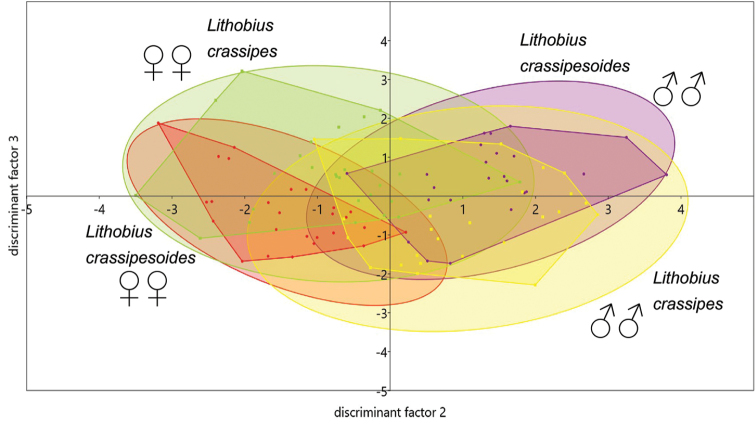
Canonical variate analysis of morphometric data showing factors two and three, using all morphometric characters of *Lithobius crassipesoides* sp. n. from Spain and *L. crassipes* from Germany.

Factor three is of no practical value and explains only a negligible part of the variance (Fig. 10).

**Figure 11.**
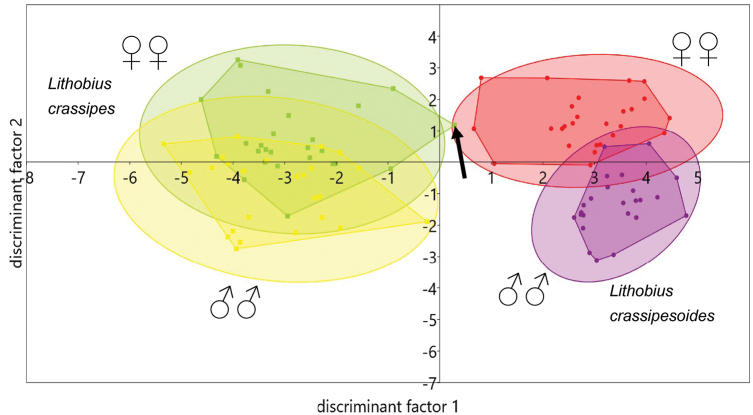
Canonical variate analysis of morphometric data of *Lithobius crassipesoides* sp. n. from Spain and *L. crassipes* from Germany, showing factors one and two. Only characters with high separating power have been included. The specimen SMNG VNR15170-2 b with an extraordinary position within the CVA is marked with an arrow.

Based on these interpretations a reduced model could be defined, which has a lower separating power but requires less measuring. Fig. 11 shows the first two discriminant factors. The parameters of the reduced model are given in Table 14.

**Table 14.** Eigenvalues and factor loading of the three extracted discriminant factors in a reduced model. Highest factor loadings marked in bold letters.

	Axis 1	Axis 2	Axis 3
Eigenvalue	9.84	1.23	0.13
Explanation of variance	87.9 %	11.0 %	1.1 %
Body length	-**0.313**	-0.080	**0.904**
Antennae length	-0.130	-0.063	0.235
Coxal pore LP15	-0.063	**0.352**	0.099
Ocelli	-0.179	-**0.278**	0.167
DaP	**0.838**	0.145	**0.393**
Vmt	-0.138	-0.008	0.033

## Discussion

### Molecular and morphological comparison of *L. crassipesoides* sp. n. and *L. crassipes*

Both molecular analysis (Fig. 1) and morphology support the hypothesis that the northern Spanish *L. crassipes*-like specimens represent a species genetically and morphologically distinct from *L. crassipes*. The two species are morphologically similar and probably closely related, but can be distinguished as both males and females. While *L. crassipes* is widely distributed in Europe, *L. crassipesoides* sp. n. is currently only known from northern Spain (Fig. 17).

### Statistical analysis

The main weakness of the morphometric statistical evaluation is the fact that the measured specimens are not identical with the sequenced specimens. This means that the possibility of having other cryptic species among the material cannot be excluded. The rejection of the normal distribution in the French samples in body length, in the German samples in head length and in samples from Spain and France in antennae length might be interpreted as a hint for more cryptic lineages.

### Canonical variate analysis

The moderate dropping of the eigenvalue of axis1 from 10.6 to 9.84 (7 %) and of axis2 from 1.49 to 1.23 (1.5 %) shows that no crucial variables have been removed when generating the reduced model. This is also visible by comparison of Fig. 9 versus Fig. 11, as the overlapping of the convex hulls changes only at a small extent. To check if the measures antennae length and head length represent discriminating power beside a size factor, their quotients had also been included in the complete model of the CVA. As visible in Table 13 head length shows only a low, negligible factor loading of -0.014, while antennae length shows a distinctly higher loading of -0.125 and the quotients AL/HL and BL/AL show lower loadings, but these are still higher than the loading of HL solely. Also a model without AL and HL was calculated, but the factor loadings of AL/HL and BL/AL did not change their values. This means that in contrast to HL AL has a discriminant value beside a size factor, and that using AL solely in a model would be the best choice.

As seen in Fig. 10 axis3 shows no clear separation for any group. This is also reflected by the low eigenvalue, explaining only 2.1 % of the overall variability in the complete and 1.1 % in the reduced model. Furthermore axis1 and axis3 show the highest factor loadings on the same variables, marked in bold letters in Table 14. For that reason it is justified to omit axis3 in further considerations.

### *Lithobius crassipes* on the Iberian Peninsula and elsewhere in Europe

Currently, all records of *L. crassipes* on the Iberian Peninsula as well as in other parts of Europe need to be carefully checked against *L. crassipesoides* sp. n., which could also be dispersed by human transport (Decker et al. 2017, Stoev et al. 2010). In the southern part of Spain more pseudo-cryptic species of the *Lithobius crassipes*-group may also exist. *L. crassipes* is formally known from the Pyrénées-Atlantiques department (French Basque country) (Iorio 2014, 2016), which is adjacent to the Spanish Navarre province. However, as mentioned above, *L. crassipesoides* sp. n. occurs only a few hundred meters or few kilometers from the French border and can be assumed to occur at least in the most western region of the French Pyrenees.

Interestingly, some authors have previously given details on morphology of Spanish *L. crassipes*. Serra (1980) described specimens of *L. crassipes* from the province of Almería as follows: 6–11 mm body length, 6–11 ocelli in 2 or 3 rows, a DaP spine on legpair 10 (or even sometimes on legpair 7) to legpair 15. Perhaps his description included specimens of both *L. crassipes* and *L. crassipesoides* sp. n. Finally, Serra (1982) mentioned the same details as above for some *L. crassipes* from the provinces Asturias and Almería but only gave the plectrotaxy of legpair 15, not the preceding legpairs.

Here we give a brief review of historic descriptions of *L. crassipes* for characters which have been found to be useful. Descriptions of *L. crassipes* elsewhere in Western Europe quoted a body length of 9–12 mm (Iorio 2008, 2010, Iorio and Labroche 2015) or 9–13 mm (Meinert 1872), up to 13.5 mm (Barber 2009); Koch (1862) gave the body length in “lines” (= lignes in French, an old measurement unit), thus his 4–4.5 lines may correspond to 9.02–10.15 mm. Manfredi (1957), in her description of *L. crassipes stictonotus* Manfredi, 1957 from southern Italy, a synonym of typical *L. crassipes* according to Bonato et al. (2016), has recorded 11.5–14 mm. Only Brölemann (1930) mentioned a small size, with 6.5–10 mm. Also, the number of ocelli ranged from 8 to 11 in two rows (Brölemann 1930), 10 to 11 in three rows (Koch 1862), 8 to 12 in two or three rows (Meinert 1872, Iorio 2008, 2010) or 9 to 13 in two or three rows (Barber 2009). Manfredi (1957) gave 12 or 13 ocelli for *L. crassipes stictonotus*. The DaP spine starts at legpair 10 (or even on legpair 7 to 9) to legpair 15 according to Brölemann (1930) and Eason (1964). Brölemann (1930) noted that the DaP spine is only sometimes present on legpair 15. Despite several hundred of specimens of *L. crassipes* having been examined from France and Germany (this study and unpublished data), no specimen with an absence of a DaP on legpair 15 was observed. Brölemann (1930) may have had specimens of *L. crassipesoides* among his material.

In Sweden, western specimens of *L. crassipes* seems to differ from the south-eastern Swedish *L. crassipes* (including also those from Denmark) as underlined by Andersson (1981). In the west, a DaP spine is only present on the last one, two or three legpairs, or a DaP may even been absent. In the south-east, *L. crassipes* have a DaP spine at least from legpair 12 to 15, but usually on more legs, 88 % from legpair 10 to 15, and it can even start on legpair 5 (Andersson 1981). The western Swedish specimens have a body length of 8–10 mm, and 8–10 ocelli; the south-eastern specimens are 9–11 mm long with 9–12 ocelli (Andersson 1981). Andersson et al. (2005) also record both forms, but without any supplementary information. We note that the south-eastern Swedish and the Danish specimens correspond well with our *L. crassipes* from France and Germany.

## Taxonomy

### Order Lithobiomorpha

#### Family Lithobiidae Newport, 1844

##### Genus *Lithobius* Leach, 1814

###### Subgenus Monotarsobius Verhoeff, 1905

Species of the subgenus Monotarsobius are characterized by short antennae and, in its modern conception, very frequently with 20 antennal articles for most members in Western Europe; the wider amplitude in this area being usually 17–23 articles, very rarely 24–25 in exception as in Spanish *L. blascoi* Eason, 1991. *Monotarsobius* always has 2 + 2 forcipular teeth, no posterior projections on tergites and tarsal articulation is missing on the first 12 or 13 legpairs (Verhoeff 1905, 1937, Brölemann 1930, Eason 1964, 1982, 1991, 1992, Serra 1980, Iorio 2008, 2010, Barber 2009).

####### Lithobius crassipesoides sp. n.

Taxon classificationAnimaliaLithobiomorphaLithobiidae

http://zoobank.org/EFB3B6F5-F82D-42AF-B1FC-6C656C0D18B7

Figures 1 , 2 , 3 , 4 , 5 , 6 , 7 , 8 , 9 , 10 , 11 , 12 , 13 , 14 , 15 , 16 , 17 , Tables 1 , 2 , 3 , 4 , 5 , 6 , 7 , 8 , 9 , 10 , 11 , 12 , 13 , 14 , 15 , 16

Lithobius crassipes – Barace and Herrera 1980: 4 (listed), 5 (records, redescription), figs 3–5. – Barace and Herrera 1982: 117 (records). – Salinas 1990: 2 (in checklist for Navarre), 59 (record), mapa 19 (map).

######## Diagnosis.

Small member (body length 6.4–11 mm) of the subgenus Monotarsobius. Antennae with 20 articles, short, 2.6 times longer than head, 1/4 of body length. 5–11 ocelli, mostly 8 or 9, in two or three rows with one larger posterior ocellus. Legs with species-specific plectrotaxy; legpair 14 and 15 thickened in both sexes, much more so in males; legpair 15 without accessory apical claw, in males with a depression in the posterior half of tibia. Female gonopod claw tridentate.

*L. crassipesoides* sp. n. differs generally from other Iberian members of *Monotarsobius* in the presence of a depression in the posterior half of the legpair 15 tibia. It differs from *L. osellai* and *L. morenoi* in having more than one row of ocelli; from *L. blascoi* in having only 20 antennal articles; and from *L. crassipes* in smaller body length, shorter antennae, lower number of ocelli, the DaP spine starting posteriorly from legpair (12) 13, and the different location and size of the male depression on legpair 15 tibia.

**Figure 12.**
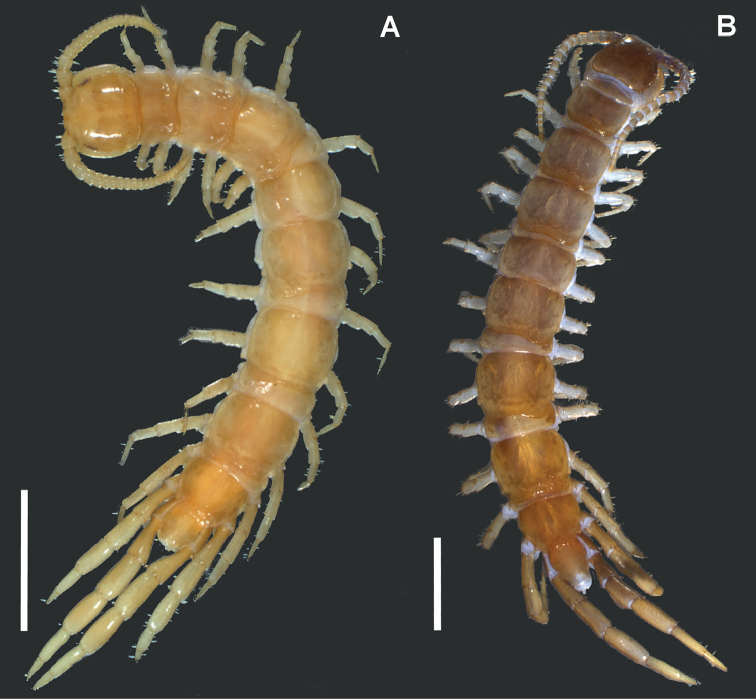
Adult males, dorsal view **A** *Lithobius crassipesoides* sp. n. (SMNG VNR14828) **B** *L. crassipes* (SMNG VNR15171). Scale bars: 2 mm (**A, B**).

**Figure 13.**
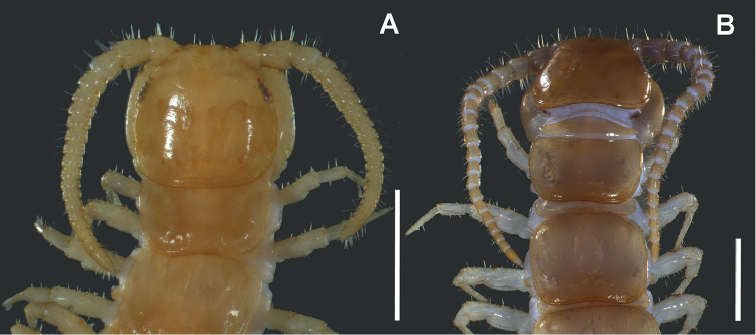
Adult males, anterior body, dorsal view **A** *Lithobius crassipesoides* sp. n. (SMNG VNR14828) **B** *L. crassipes* (SMNG VNR15171). Scale bars: 0.5 mm (**A, B**).

**Figure 14.**
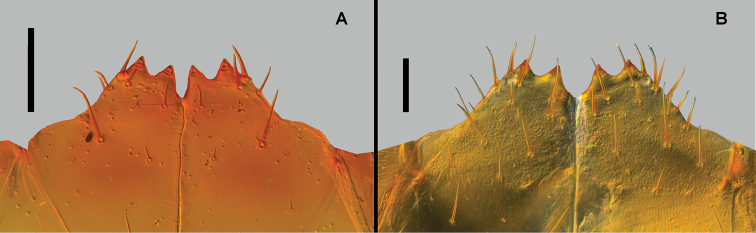
Coxosternum, ventral view **A** *Lithobius crassipesoides* sp. n. (MZNA 138944) **B** *L. crassipes* (SMNG VNR015171). Scale bars: 0.1 mm (**A, B**).

######## Etymology.

Derived from the morphological similarity to *Lithobius crassipes*.

######## Material examined.

*Holotype.* Spain: Navarre province: Leitza, Ariz Mendiak, between area “Ustarleku” and “Karobieta”, above side stream to Gorriztaran; loamy and calcareous soil, 43.0778°N, 1.8775°W, 615 m a.s.l., 20 April 2009, leg. FT2009, grove of *Castanea*, pollard trees on the slope with *Ranunculus ficaria*, *Daphne*, *Helleborus*, *Salvia*, *Rubus*, *Lathrea*; under leaves and bark of rotten trunk, 1 ♂ (SMNG VNR14744-4 d).

*Paratype.* Spain: Navarre province: Same data as holotype, 1 ♂ (SMNG VNR16739-1 b), 1 ♀ (SMNG VNR14744-4 b), 1 ♀ (SMNG VNR14752-1 c); Spain: Gipuzkoa province, Sierra de Aralar, Beasain, road from Lazkao to Etxarri-Aranaz, Pass Puerto de Lizarrusti, forest of *Fagus* on the slope, under stones and in leaf litter, 42.9614°N, 2.0983°W, 690 m a.s.l, 21 April 2009, leg. FT2009, 1 ♂: (SMNG VNR14764-3).

*Other material examined.* **Spain: Navarre province**: Leitza, Ariz Mendiak, between area “Ustarleku” and “Karobieta”, above side stream to Gorriztaran; loamy and calcareous soil, 43.0778°N, 1.8775°W, 615 m a.s.l., 20 April 2009, leg. FT2009, grove of *Castanea*, pollard trees on the slope with *Ranunculus ficaria*, *Daphne*, *Helleborus*, *Salvia*, *Rubus*, *Lathrea*; under leaves and bark of rotten trunk, 2 ♂♂, 3 ♀♀, 1 juv. ♂ (SMNG VNR14744-4), 1 ♂, 1 juv. ♂ (SMNG VNR16739-1), 1 ♀ (SMNG VNR14746-5), 1 ♀ (SMNG VNR17128-8); 100 meters down to a meadow along the way, 1 ♀ (SMNG VNR17126-4); *Alnus* wood along stream, under bark of standing dead wood, 2 ♀♀, 1 juv. ♀ (SMNG VNR14752-1); with *Carpinus betulus, Corylus, Fagus, Sambucus*, 1 ♀ (SMNG VNR17129-3). – Leitza, Ariz Mendiak, between area “Ustarleku” and “Karobieta”, next to road NA 4150 to Goizueta; on a slope with *Fagus*, *Corylus*, under a dead, mossy tree trunk, 43.09092°N, 1.86613°W, 593 m a.s.l., 20 April 2009, leg. FT2009, 2 ♀♀ (SMNG VNR17131-3). – Sierra de Aralar, south of Errazkin, north of area “Axkarateko Malkorra”, young *Fagus* and old big *Quercus*, *Ilex*, *Ruscus*, *Hedera helix*, in leaf litter, 42.9972°N, 1.9703°W, 630 m a.s.l., 21 April 2009, leg. FT2009, 2 juv. ♂♂ (SMNG VNR14757-6), 1 ♂, 1 ♀ (SMNG VNR14758-3; SMNG VNR14813-1), 1 ♂, 1 ♀ (SMNG VNR17133-9). – Sierra de Aralar, south Baraibar, on road NA-7510 to Santuario de San Migel, at area “Izáin”, karst area with deep grykes and bare limestone rocks, *Fagus* woodland and some *Crataegus* bushes, in leaf litter, partly sieved out, 42.9714°N, 1.9384°W, 790 m a.s.l., 22 April 2009, leg. FT2009, 1 ♀ (SMNG VNR14770-12), 1 ♀ (SMNG VNR14771-6). – Sierra de San Miguel, mountain point Artxueta at radio mast, karst area with bare limestone rocks, grove of low growing *Fagus*, in litter and under bark, 42.9525°N, 1.9668°W, 1300 m a.s.l., 22 April 2009, leg. FT2009, 3 ♂♂, 1 ♀ (SMNG VNR14773-1). – Sierra de Urbasa, road NA-718 from Olazi / Olazagutía to Estella, at the end of the hairpin curves, under the bark of a very large old beech with a lot of moos; limestone, 42.86031°N, 2.18055°W, 888 m a.s.l., 23 April 2009, leg. FT2009, 1 ♂, 1 ♀ (SMNG VNR17135-4). – Sierra de Urbasa, 1 km east of road junction to road NA-7182 at site “Bentakaita”, *Fagus* forest with *Prunus*, *Corydalis*, *Mercurialis*, *Erythronium*; in leaf litter, 42.8540°N, 2.1595°W, 890 m a.s.l., 23 April 2009, leg. FT2009, 1 ♂ (SMNG VNR16734-1), 1 ♂ (SMNG VNR17136-4). – Urroz, hayedo [beech forest], 25 September 1995, leg. Javier Sáenz de Cabezón, 1 ♀ (MZNA MZ-19951125 a). – Imbuluzqueta, tocón [stub], 21 February 1993, 2 ♂♂, 4 ♀♀ (MZNA MZ-19931121a). – Eguaras, Vedado de, *Quercus coccifera*, 10 January 1980, leg. R. Jordana, 1 juv. ♀ (MZNA VE1041AS). – Irati, suelo de hayedo [floor of beech forest], 17 September 1982, leg. J. Barace, 1 ♀ (MZNA MZ-19820917, Salinas 1990). – Velate, hayedo [beech forest], 25 September 1995, leg. Javier Sáenz de Cabezón, 1 ♂ (MZNA MZ-19951125). – Lagos de Urroz, hayedo [beech forest], 25 September 1995, leg. Javier Sáenz de Cabezón, 1 ♀ (MZNA MZ-19951125 a). – Tirapegui, tocón [stub], 21 February 1993, 3 ♂♂, 1 ♀ (MZNA MZ-19931121 b). – Aquerreta, corteza [bark], 15 January 1994, 1 ♂, 6 ♀♀ (MZNA MZ-19940119a). – Quinto Real, 29 June 1977, 1 juv. ♀ (MZNA MZ-19770629d, Barace and Herrera 1980). – Quinto Real, carretera [road], 11 May 1977, leg. R. Jordana, 1 ♀ (MZNA MZ-19770511d, Barace and Herrera 1980). – Quinto Real, tocón [stub], 4 August 1977, leg. Vierna, 4 ♂♂ (MZNA MZ-19770804e, Barace and Herrera 1982). – Quinto Real, carretera [road], 11 May 1977, 1 ♂ (MZNA MZ-19770511d, Barace and Herrera 1980). – Quinto Real, pinar [pine forest], 14 September 1977, 1 ♂ (MZNA MZ-19770914, Barace and Herrera 1980). – Quinto Real, 24 November 1976, leg. Labiano, 1 ♂ (MZNA MZ-19761124a, Barace and Herrera 1980). – Quinto Real, 29 June 1977, leg. Monreal, 1 ♂ (MZNA MZ-19770629d, Barace and Herrera 1980). – Quinto Real, pinar [pine forest], 14 September 1977, 1 ♀ (MZNA MZ-19770914, Barace and Herrera 1980). – Quinto Real, pinar [pine forest], 14 September 1977, 1 ♀ (MZNA MZ-19770914, Barace and Herrera 1980). – Quinto Real, corteza de haya [bark of beech], 3 September 1977, leg. J. Barace, 1 ♂ (MZNA MZ-19770903 d, Barace and Herrera 1980). – Quinto Real, pinar [pine forest], 14 September 1977, 1 ♂ (MZNA MZ-19770914, Barace and Herrera 1982). – Quinto Real, corteza [bark], 29 June 1977, 1 ♂ (MZNA MZ-19770629 e, Barace and Herrera 1980). – Quinto Real, corteza [bark], 29 June 1977, 1 (juv.?) ♀ (MZNA MZ-19770629 e, Barace and Herrera 1980). – Quinto Real, tocón [stub], 4 August 1977, 1 ♀ (MZNA MZ-19770804 f, Barace and Herrera 1980). – Quinto Real, tocón [stub], 16 March 1977, leg. Labiano, 1 ♀ (MZNA MZ-19770316 b, Barace and Herrera 1980). **Spain: Gipuzkoa province**: Sierra de Aralar, Beasain, road from Lazkao to Etxarri-Aranaz, west of the Pass Puerto de Lizarrusti, forest of *Fagus*, in leaf litter, 42.9572°N, 2.1122°W, 550 m a.s.l., 21 April 2009, leg. FT2009, 1 ♂ (SMNG VNR14763-9), 1 juv. ♂ (SMNG VNR14763-11). – Natural Park Aizkorri-Aratz, near Onati; close to the monastery Arantzazu, beechwood in limestone with *Ilex, Fagus, Rubus fructicosus, Hedera, Larix, Helleborus*, under bark of a dead tree trunk, 42.97766°N, 2.38989°W, 850 m a.s.l., 24 April 2009, leg. FT2009, 1 ♀ (SMNG VNR17139-5). – Natural Park Aizkorri-Aratz, Sierra de Urquilla, Montes de Altzania, south of mountain chain Aikorriko Mendikatea, south west of Mountain Aitzgorri, northwest Zumarraundi, high plain Alizania, north of Portua Zarra, karst plain with small growing trees of *Fagus* and open grasslands, 42.9354°N, 2.3298°W, 1160 m a.s.l., 24 April 2009, leg. FT2009, 1 ♂, 1 ♀, 1 juv. ♂, 1 juv. ♀ (SMNG VNR14786-3), 1 ♂, 1 ♀ (SMNG VNR14828-3), 1 ♂ (SMNG VNR14791-5). – Natural Park Aizkorri-Aratz, Sierra de Urquilla, near Zumaraundi, deep doline with creek discharge near cave entry; much leaf litter; sieved from *Fagus* leaves 42.9241°N, 2.3224°W, 980 m a.s.l., 24 April 2009, leg. FT2009, 1 juv. ♂ (SMNG VNR14793-5).

######## Description.

*Habitus.* Slightly fusiform, widest around tergite 10 (Fig. 12A).

*Colour.* General body colouration varies from pale and light buff (most individuals), through yellow ochre to tawny olive (Fig. 12A). Mostly last third of the body and usually also the head a little darker. Head light yellow ochre, sometimes darker in front and around the ocellar area. Last antennal articles yellowish. Some or all tergites more or less darker on posterior margin. Most specimens are paler than *L. crassipes*.

*Length.* 6.4–11.0 mm (Fig. 2, Table 2).

*Head.* Head roundish, mostly as broad as long or little broader than long and head broader or as broad as T5. Head length 0.6–1.08 mm (Fig. 3, Table 4).

*Antennae*. 20 antennal articles, short and stout, 1.6–3.2 mm long, 2.6 times longer than head (Figs 4, 13A, Table 6).

*Ocelli*. 5 to 11 ocelli on each side of the head, mostly 8 or 9, rarely 5 or 11, arranged in two or three rows (Fig. 5, Table 8), posteriorly with one greater, longitudinally oval ocellus, clearly separated from the others. The most common arrangements (n = 47) are 1 + 4, 3 (21 %), 1 + 4, 3, 1 (14 %) and 1 + 4, 3, 2 (14 %). There is no significant difference between males and females in number and arrangement. In 54 % the number of ocelli differs between right and left side in one or two ocelli.

*Coxosternum*. Anterior border with 2+2 teeth, upper part slender, acuminate, lateral borders without shoulders. Middle notch narrow to moderate width. Sometimes coxosternum slenderer and the middle notch narrower (Fig. 14A).

*Tergites*. Surface slightly rough, glossy. Posterior border of T1 feebly concave or straight, T3 to T5 feebly concave, T8 to T15 distinctly concave, T16 feebly to distinctly concave. Posterior angles of T9, T11 and T13 mostly obtuse or rounded with no trace of lobes or triangular projections.

*Legs.* Tarsus and metatarsus fused on legpair 1 to 11. On legpair 12 and 13 the tarsal-metatarsal articulation is indistinct. Penultimate and ultimate legpairs (14, 15) are densely covered with pores. Last two legpairs are thickened in both sexes, much more so in males. Without accessory apical claw on legpair 15.

**Figure 15.**
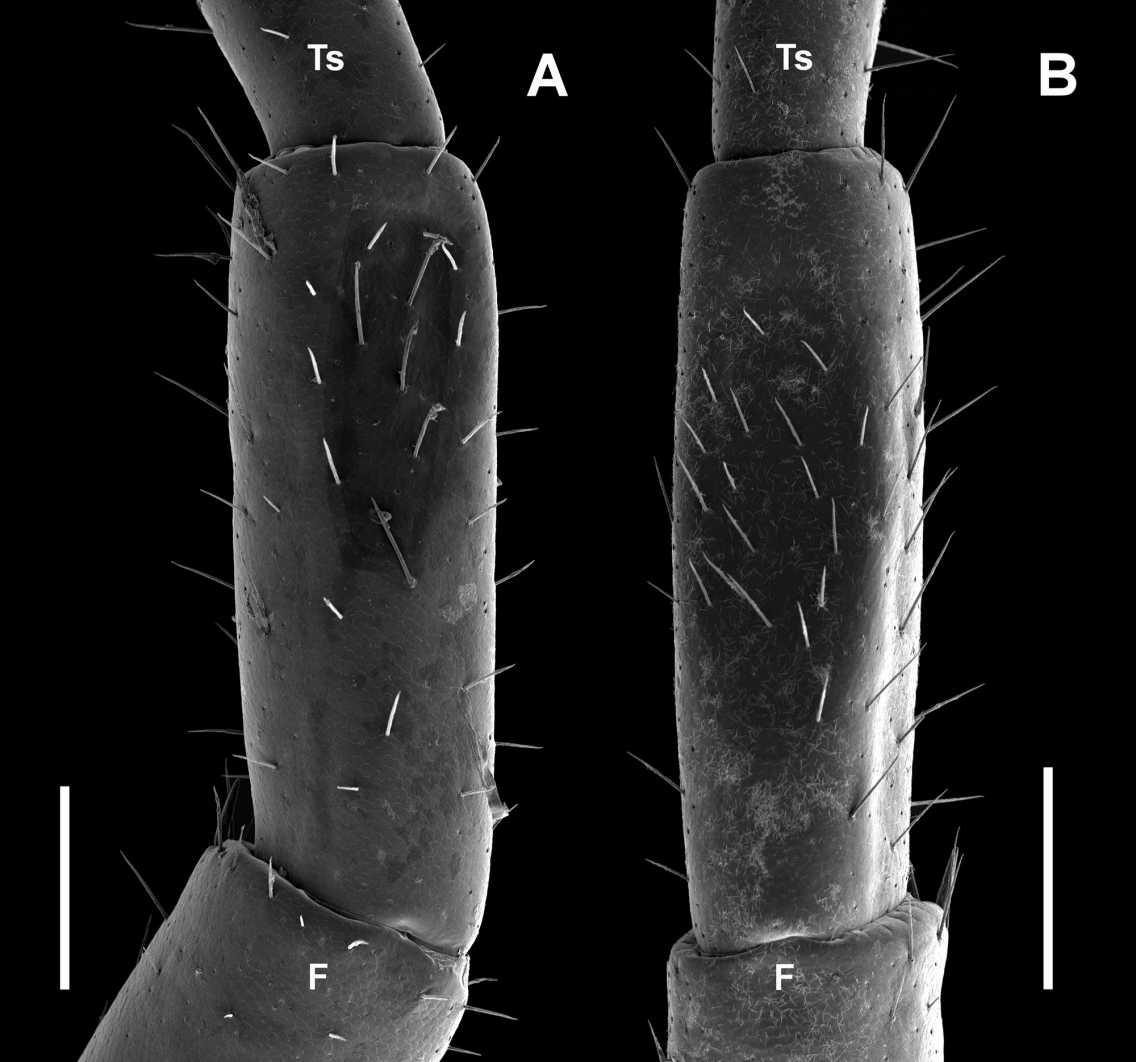
Tibia of male legpair 15, dorsal view **A** *Lithobius crassipesoides* sp. n. (SMNG VNR14773), right leg **B** *L. crassipes* (SMNG VNR10335), left leg. Abbreviations: F = femur; Ts = tarsus. Scale bars: 0.2 mm (**A, B**).

Legpair 15 tibia of males with a more setiferous depression (fossa), which is distinct and well-developed in specimens in later developmental stages (Fig. 15A), in younger developmental stages only indicated. The depression starts more or less in the half of the tibia (55–75 % of tibia length) and reaches nearly up to the end of the tibia (88–93 % of tibia length) and has a relative length of about 30 to 40 % of tibia (n = 28).

*Coxal pores.* Round, 2–4 (sometimes 5) pores on each coxa (Fig. 6, Table 9). Mostly 2, 3, 3, 2 or 3, 3, 3, 2 (coxae 15–12) with the highest observed number of 4, 5, 4, 3 in a female (Table 15).

*Plectrotaxy*. The plectrotaxy of legs of *L. crassipesoides* sp. n. is given in Table 16. It differs from *L. crassipes* in the absence of a DaP spine up to legpair 11, DaP very rarely present on legpair 12, rarely present on legpair 13 to 14, while almost always present in *L. crassipes* on legpair 10 to 15, frequently on legpair 9, rarely also on legpair 7 and 8 or only on legpair 11 to 15 (Table 17).

**Table 16.** Plectrotaxy of *Lithobius crassipesoides* sp. n. (n = 11 ♂♂ and 11 ♀♀). In brackets spines absent in more than 50 % of individuals.

Legpair	Ventral					Dorsal				
	C	t	P	F	T	C	t	P	F	T
**1**	–	–	(p)	a m(p)	m	–	–	m p	a(p)	a
**2**	–	–	(p)	a m(p)	(a)m	–	–	m p	a p	a(p)
**3**	–	–	(p)	a m(p)	(a)m	–	–	m p	a p	a(p)
**4**	–	–	(p)	a m(p)	(a)m	–	–	m p	a p	a(p)
**5**	–	–	(p)	a m(p)	(a)m	–	–	m p	a p	a(p)
**6**	–	–	(p)	a m(p)	(a)m	–	–	m p	a p	a(p)
**7**	–	–	(p)	a m(p)	(a)m	–	–	m p	a p	a(p)
**8**	–	–	(p)	a m(p)	a m	–	–	m p	a p	a(p)
**9**	–	–	(p)	a m p	a m	–	–	m p	a p	a p
**10**	–	–	(mp)	a m p	a m	–	–	m p	(a)p	a p
**11**	–	–	(m)p	a m p	a m	–	–	m p	(a)p	a p
**12**	–	(m)	(a)mp	a m p	a m	(a)	–	(a)mp	p	(a)p
**13**	–	m	a m p	a m p	a m	a	–	(a)mp	p	p
**14**	–	m	a m p	a m p	(a)m	a	–	(a)mp	p	(p)
**15**	–	m	a m p	a m(p)	–	a	–	a m p	(p)	–

*Male gonopods.* Uni-articulated.

**Figure 16.**
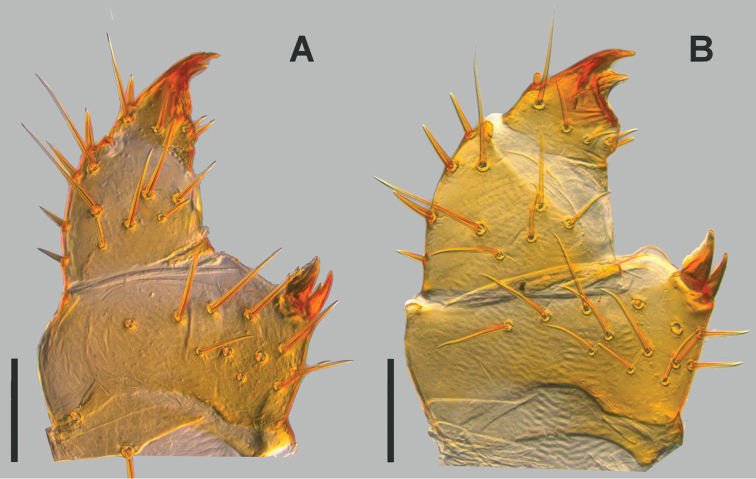
Left female gonopod, ventral view **A** *Lithobius crassipesoides* sp. n. (SMNG VNR14828) **B** *L. crassipes* (SMNG VNR15171). Scale bars: 0.1 mm (**A, B**).

*Female gonopods.* Basal article with two conical spurs on each side, their apical edge serrated (Fig. 16A). With 5–9 ventrolateral setae with nearly the same length. No dorsomedial setae. One specimen (SMNG VNR14770) with two minute setae. Article II with 4–6 dorsolateral setae, stout, straight and fairly long, evenly distributed over the whole length of the article. Ventrolaterally with 4–6 setae without characteristic arrangement. Article III with 1 dorsolateral seta, 1 ventrolateral, 1 ventral and 2 ventromedial setae. Claw tridentated, dorsal denticle longer than the ventral denticle. At high magnification (SEM) with distinct pores of glandulae.

######## Distribution.

**Figure 17.**
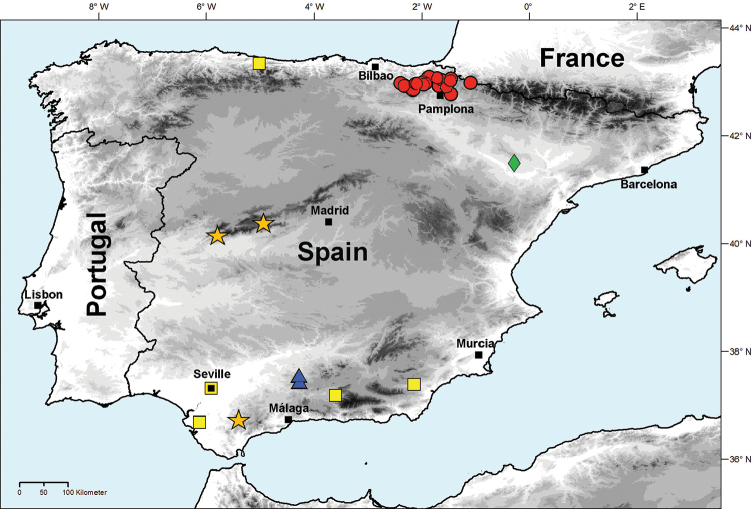
Distribution map of Lithobius (Monotarsobius) on the Iberian Peninsula: *Lithobius blascoi* = diamond; *L. crassipes* = square; *L. crassipesoides* sp. n. = circle; *L. morenoi* stat. n. = triangle; *L. osellai* = star.

So far only known from Navarre and Gipuzkoa provinces, northern Spain (Fig. 17). Some records are only a few kilometers from the French border. *L. crassipesoides* sp. n. is therefore expected also in the Western Pyrenees in France.

######## Habitat.

The species was mostly found in the leaf litter and under the bark of dead wood in mountain deciduous forests from 550 to 1300 m a.s.l.

######## Remarks.

*L. crassipes* was recorded from the Navarre region from Irati (Salinas 1990) and several locations near Quinto Real (Barace and Herrera 1980, 1982). Barace and Herrera (1980) also gave a brief description of 12 males and eight females. Most of the material studied by Barace and Herrera (1980, 1982) and Salinas (1990) was available for re-examination at MZNA (38 specimens) and was confirmed to be *L. crassipesoides* sp. n. Therefore, all records of Barace and Herrera (1980, 1982) and Salinas (1990) of *L. crassipes* are hereby assigned to *L. crassipesoides* sp. n. Finally, as mentioned above, the description of Serra (1980) for *L. crassipes* is possibly composite and perhaps partially concerns *L. crassipesoides* sp. n.

####### Lithobius (Monotarsobius) crassipes

Taxon classificationAnimaliaLithobiomorphaLithobiidae

L. Koch, 1862

Figs 1 , 2 , 3 , 4 , 5 , 6 , 7 , 8 , 9 , 10 , 11 , 12 , 13 , 14 , 15 , 16 , 17 , Tables 1 , 2 , 3 , 4 , 5 , 6 , 7 , 8 , 9 , 10 , 11 , 12 , 13 , 14 , 15 , 17

Lithobius crassipes L. Koch 1862: 71–72, table II 31
Lithobius crassipes – Meinert 1872: 341 (record). – Attems 1927: 238 (record). – Attems 1952: 346 (record). – Demange 1958: 39 (in key). – Serra 1980: 294-297 (record, description). – Serra 1982: 45–47, figs a–c (record, description), 49 (in key). – Garcia Ruiz 2015: 9 (in checklist for Spain).

######## Description.

*Habitus.* Slightly fusiform, widest around tergite 10 (Fig. 12B).

*Colour.* General body colouration varies from pale horn colour to cream colour and to yellow ochre (Fig. 12B) or rarely with very light reddish tint.

*Length.* 7.6–13.2 mm (Fig. 2, Table 2).

*Head.* Head roundish, mostly as broad as long or little broader than long and head broader or as broad as T5. Head length 0.8–1.12 mm (Fig. 3, Table 4).

*Antennae*. 20 antennal articles, short and stout, 2–4 mm long, 3.2 times longer than head, length (Figs 4, 13B, Table 6).

*Ocelli*. The German specimens have 8–12, the French 7–15 ocelli on each side of the head, mostly 9–11 (Fig. 5, Table 8), posteriorly with one greater, longitudinally oval ocellus, clearly separated from the others. In 90 % of all specimens (n = 174) they are arranged in three rows with a single larger ocellus posteriorly. The most common arrangements are in Germany (n = 52) 1+432 (43 %), 1+431 (11 %), 1+433 (10 %) and in France (n = 125) 1+432 (47 %), 1+431 (19 %), 1+442 (15 %). In nearly 60 % of individuals (both Germany and France) the number differs between right and left side of head, mostly only in one ocellus.

*Coxosternum*. Anterior border with 2+2 teeth, upper part slender, acuminate, lateral borders without shoulders. Middle notch narrow to moderate width. Sometimes coxosternum broader and the middle notch broader (Fig. 14B).

*Tergites*. Surface slightly rough, glossy. Posterior border of T1 feebly concave or straight, T3 to T5 feebly concave, T8 to T15 distinctly concave, T16 feebly to distinctly concave. Posterior angles of T9, T11 and T13 mostly obtuse or rounded with no trace of lobes or projections.

*Legs.* Tarsus and metatarsus fused on legpair 1 to 11. On legpair 12 and 13 the tarsal-metatarsal articulation is indistinct. Penultimate and ultimate legpairs (14, 15) are densely covered with pores. Last two legpairs are thickened in both sexes, much more so in males. Without accessory apical claw on legpair 15.

Legpair 15 tibia of males with a more setiferous depression (fossa), which is distinct and well-developed in specimens in later developmental stages (Fig. 15B), in younger developmental stages only indicated. The depression starts in the first third of the tibia (20–40 % of tibia length) and reaches nearly up to the end of the tibia (80–90 % of tibia length) and has a relative length of about 40 to 50 % of tibia (n = 85).

*Coxal pores.* Round, 2–4 (sometimes 5) pores on each coxa (Fig. 6, Table 9). Mostly 2, 3, 3, 2 or 3, 3, 3, 2 (coxae 15–12) with the highest observed number of 4, 5, 5, 4 in a female (Table 15).

**Table 15.** Coxal pores in *Lithobius crassipesoides* sp. n. and in German *L. crassipes*. Sequence from legpair 15–12.

	*Lithobius crassipesoides* sp. n.		*Lithobius crassipes* (Germany)	
	♂♂	♀♀	♂♂	♀♀
**n**	31	31	26	26
**Variants of arrangements**	7	7	5	14
**Highest number**	2432, 2342	4543	3443	4554
**Common arrangements**	2332 (71 %)	3332 (45 %)	2332 (42 %)	2332 (15 %)
		2332 (32 %)	3332 (39 %)	3443 (15 %)

*Plectrotaxy*. The plectrotaxy of legs of *L. crassipes* is given in Table 17.

**Table 17.** Plectrotaxy of German *Lithobius crassipes* (n = 11 ♂♂ and 11 ♀♀). In brackets spines absent in more than 50 % of individuals.

Legpair	Ventral					Dorsal				
	C	t	P	F	T	C	t	P	F	T
**1**	–	–	–	a m	m	–	–	m p	a(p)	a
**2**	–	–	–	a m	(a)m	–	–	m p	a p	a(p)
**3**	–	–	–	a m	(a)m	–	–	m p	a p	a(p)
**4**	–	–	–	a m(p)	(a)m	–	–	m p	a p	a p
**5**	–	–	–	a m(p)	(a)m	–	–	m p	a p	a p
**6**	–	–	–	a m(p)	a m	–	–	m p	a p	a p
**7**	–	–	–	a m(p)	a m	–	–	(a)mp	a p	a p
**8**	–	–	–	a m(p)	a m	–	–	(a)mp	a p	a p
**9**	–	–	(mp)	a m p	a m	–	–	a m p	a p	a p
**10**	–	–	m(p)	a m p	a m	–	–	a m p	a p	a p
**11**	–	–	m p	a m p	a m	–	–	a m p	a p	a p
**12**	–	–	(a)mp	a m p	a m	(a)	–	a m p	p	p
**13**	–	(m)	a m p	a m p	a m	a	–	a m p	p	p
**14**	–	m	a m p	a m p	m	a	–	a m p	p	(p)
**15**	–	m	a m p	a m	–	a	–	a m p	(p)	–

*Male gonopods.* Uni-articulated.

*Female gonopods.* Basal article with two conical spurs on each side, their apical edge serrated (Fig. 16B). With 6–13 (mostly 8) ventrolateral setae with nearly the same length. No dorsomedial setae. Article II with 3–4 (mostly 3) dorsolateral setae, stout, straight and fairly long, evenly distributed over the whole length of the article. Ventrolaterally with 4–6 (mostly 6) setae without characteristic arrangement. Article III with 1 dorsolateral seta, 1 ventrolateral, 1 ventral and 2 ventromedial setae. Claw tridentated, dorsal denticle longer than the ventral denticle. At high magnification (SEM) with distinct pores of glandulae.

######## Distribution.

Widespread in Europe. The species has been recorded from several more or less precise Spanish localities (Fig. 17): Granada (Meinert 1872, Attems 1927); Cerro del Mirador, Sevilla (Attems 1952); Av. del Roquer, Chiribel, Albox, Almería (Serra 1980, 1982); Mestas de Con, Asturias (Serra 1982).

All current records of *L. crassipes* on the Iberian Peninsula are doubtful and need further verification (see discussion above). Some records from the literature have been assigned here to *L. crassipesoides* sp. n. or *L. morenoi*.

####### Lithobius (Monotarsobius) morenoi

Taxon classificationAnimaliaLithobiomorphaLithobiidae

Garcia Ruiz & Baena, 2014 stat. n.

Fig. 17

Lithobius crassipes morenoi Garcia Ruiz and Baena, 2014: 58–61, fig. 1, tables I–II.
Lithobius crassipes morenoi – Serrra 1982: (record). – Garcia Ruiz and Baena 2014: 61 (discussion). – Garcia Ruiz 2015: 9 (in checklist for Spain).

######## Distribution.

Known from two caves (Sima LQ-14, Abuchite, Luque; Sima de la Sierrezuela/Sima Fuente del Francés, Carcabuey) in Andalusia and from Jerez de la Frontera (Cádiz), Spain (Fig. 17).

######## Remarks.

This subspecies was described by Garcia Ruiz and Baena (2014) based on specimens from two caves in the Cordoba province in Andalusia. It clearly differs from *L. crassipes crassipes* in the low number of ocelli (only three), the enlarged Tömösváry’s organ, and the absence of some spines, particularly of Vmt, VaF, and DaH on legpair 15. Garcia Ruiz and Baena (2014) also discuss the record of *L. crassipes* by Serra (1982) coming from Cv. de las Motillas, Jerez de la Frontera (Cádiz), which was also considered by them as probably representing *L. crassipes morenoi*. The specimens of Serra (1982) have the same morphological peculiarities regarding number of ocelli (three), Tömösváry’s organ and modification of male legpair 15 tibia as the subspecies *L. c. morenoi*. All records from *L. c. morenoi* are from the same geographical area in Andalusia, localized near the cities of Seville and Cordoba. Therefore, we are elevating *L. c. morenoi* species status and assigning the Jerez de la Frontera records of *L. crassipes* to *L. morenoi* stat. n.

####### Lithobius (Monotarsobius) blascoi

Taxon classificationAnimaliaLithobiomorphaLithobiidae

Eason, 1991

Fig. 17

Lithobius blascoi Eason 1991: 179–183, figs. 1–6, table 1
Lithobius blascoi – Garcia Ruiz and Baena 2014: 56 (listed). – Garcia Ruiz 2015: 9 (in checklist for Spain).

######## Distribution.

So far only known from the type locality Pina de Ebro near Zaragoza, Spain (Fig. 17).

####### Lithobius (Monotarsobius) osellai

Taxon classificationAnimaliaLithobiomorphaLithobiidae

Matic, 1968

Fig. 17

Lithobius osellai Matic 1968: 123–125, fig. 4
Lithobius osellai – Serrra 1982: 47 (record, description), 49 (in key). – Garcia Ruiz and Baena 2014: 56 (listed). – Garcia Ruiz 2015: 9 (in checklist Spain).
Lithobius cf. osellai – Voigtländer and Reip 2013: 235, tab. 1, figs 27–30 (record, description).

######## Distribution.

In Spain (Fig. 17) known from two localities in the Sierra de Gredos, in central Spain (Matic 1968, Serra 1982), and one uncertain record from the Sierra de Grazalema, Andalusia (Voigtländer and Reip 2013). There is also one doubtful record from Malta (Kime 2003).

###### Key to the species of Lithobius (Monotarsobius) of the Iberian Peninsula

Key is valid for adult specimens only.

**Table:** 

1	3 or 4 ocelli arranged in one row only	**2**
–	6–13 ocelli arranged in two or three rows	**3**
2	Ventral plectrotaxy of LP15 = --, m, amp, -m-, ---. VaP present from LP13 to LP15, VpF from LP8 to LP14, VaT from LP7 to LP13 and DaP from LP10 to LP15. Male LP15 tibia with a weak depression on the dorsal side	***L. morenoi* Garcia Ruiz & Baena, 2014 stat. n.**
–	Ventral plectrotaxy of LP15 = --, m, -mp, -m-, -m-. No VaP, VpF, VaT and DaP spines on legs	***L. osellai* Matic, 1968**
3	Antennae with 22 to 23 (rarely 24, unusually 25) articles. 6–7 ocelli arranged in two rows (usually 1 + 3, 3). Angular shoulders on each side of the lateral teeth on the forcipular coxosternite. No VaP, VaF, VpF, DaP nor DpT spines on legs. Male with a dorsal wart near the extremity of the LP15 femur and a weak sulcus in the distal two thirds of LP15 tibia	***L. blascoi* Eason, 1991**
–	Antennae with usually 20 articles (18–21). 6–13 ocelli arranged in two or three rows. No angular shoulders on each side of the lateral forcipular teeth. VaP at least exist on LP15, VaF at least from LP12 to LP15, VpF at least from LP13 to LP14, DaP at least on LP15 (or very rarely lacking), DpT at least on LP12 and/or LP13. Male with a depression on dorsal side of LP15 tibia	**4**
4	Body usually 6–11 mm long. Usually, DaP starts on LP14 or on LP15, exceptionally from LP12, rarely from LP13 or totally lacking. Male dorsal depression of LP15 tibia shorter; starts in the posterior half of LP15 tibia (Fig. 15A)	***L. crassipesoides* Voigtländer, Iorio, Decker & Spelda, sp. n.**
–	Body 8.5–12 mm long (extreme values 7.9 and 13.2). Usually, DaP starts from LP7 to LP11 until LP15, very rarely from LP12 or LP13. Male dorsal depression of LP15 tibia longer, starts in the first third of LP15 tibia (Fig. 15B)	***L. crassipes* L. Koch, 1862**

## Supplementary Material

XML Treatment for Lithobius crassipesoides

XML Treatment for Lithobius (Monotarsobius) crassipes

XML Treatment for Lithobius (Monotarsobius) morenoi

XML Treatment for Lithobius (Monotarsobius) blascoi

XML Treatment for Lithobius (Monotarsobius) osellai

## Supplementary materials

Supplementary material 1 Full data of investigated specimens of *Lithobius crassipes* Data type: Occurence and collection Explanation note: Information on locality, date, collector, collection, sex of investigated specimens of *Lithobius crassipes*. This dataset is made available under the Open Database License (http://opendatacommons.org/licenses/odbl/1.0/). The Open Database License (ODbL) is a license agreement intended to allow users to freely share, modify, and use this Dataset while maintaining this same freedom for others, provided that the original source and author(s) are credited.Karin Voigtländer, Etienne Iorio, Peter Decker
